# PD-1 and PD-L1 inhibitors in cold colorectal cancer: challenges and strategies

**DOI:** 10.1007/s00262-023-03520-5

**Published:** 2023-10-13

**Authors:** Ke Xin Lin, Alexandra C. Istl, Douglas Quan, Anton Skaro, Ephraim Tang, Xiufen Zheng

**Affiliations:** 1 Department of Pathology, University of Western Ontario, London, ON N6A 5A5 Canada; 2 Department of Physiology and Pharmacology, University of Western Ontario, London, Canada; 3 Division of Surgical Oncology, Medical College of Wisconsin, Milwaukee, WI USA; 4 Department of Surgery, University of Western Ontario, London, ON N6A 5A5 Canada; 5 Department of Oncology, University of Western Ontario, London, ON N6A 5A5 Canada; 6 Department of Microbiology & Immunology, University of Western Ontario, London, ON N6A 5A5 Canada; 7 Lawson Health Research Institute, London, ON N6A 5A5 Canada; 8 Temerty Faculty of Medicine, University of Toronto, Toronto, ON Canada

**Keywords:** PD-L1, PD-1, Immune checkpoint inhibitor, Colorectal cancer, Immune therapy

## Abstract

Colorectal cancer (CRC) is the second most common cause of cancer mortality, with mismatch repair proficient (pMMR) and/or microsatellite stable (MSS) CRC making up more than 80% of metastatic CRC. Programmed death-ligand 1 (PD-L1) and programmed death 1 (PD-1) immune checkpoint inhibitors (ICIs) are approved as monotherapy in many cancers including a subset of advanced or metastatic colorectal cancer (CRC) with deficiency in mismatch repair (dMMR) and/or high microsatellite instability (MSI-H). However, proficient mismatch repair and microsatellite stable (pMMR/MSS) cold CRCs have not shown clinical response to ICIs alone. To potentiate the anti-tumor response of PD-L1/PD-1 inhibitors in patients with MSS cold cancer, combination strategies currently being investigated include dual ICI, and PD-L1/PD-1 inhibitors in combination with chemotherapy, radiotherapy, vascular endothelial growth factor (VEGF) /VEGF receptor (VEGFR) inhibitors, mitogen-activated protein kinase (MEK) inhibitors, and signal transducer and activation of transcription 3 (STAT3) inhibitors. This paper will review the mechanisms of PD-1/PD-L1 ICI resistance in pMMR/MSS CRC and potential combination strategies to overcome this resistance, summarize the published clinical experience with different combination therapies, and make recommendations for future avenues of research.

**Supplementary Information:**

The online version contains supplementary material available at 10.1007/s00262-023-03520-5.

## Introduction

The development of immunotherapeutic drugs has led to significant improvements in overall and progression-free survival for many patients with cancer [1–6]. For colorectal cancer (CRC) in particular, immunotherapy has demonstrated significant benefit in the metastatic setting for a subset of patients [3, 4, 6, 7]. Programmed death 1 (PD-1) is an immune checkpoint receptor mainly expressed in T cells, B cells, natural killer cells (NKs), and myeloid-derived suppressor cells (MDSCs) [8–12]. It binds to its ligands, programmed death-ligand 1 and 2 (PD-L1/2), which are expressed on antigen presenting cells and cancerous cells [10–13]. The interaction between PD-1 and PD-L1/2 induces T cell exhaustion, inhibits T cell activation and cytotoxic activity, and transforms T effector cells to regulatory T cells (Treg) [10–13]. As such, blockade of the PD-1/PD-L1/2 pathway can enhance T cell anti-tumor activity and thereby immune control and killing abilities against cancerous cells. The introduction of immunotherapy with immune checkpoint inhibitors (ICIs) targeting PD-1 and PD-L1 has revolutionized management of certain cancers, transforming short-term responses into durable clinical benefits [4, 5, 13, 14]. However, tumors that do not elicit an immune response, so called ‘cold’ tumors, exhibit resistance to this strategy [6, 7, 13, 15, 16]. Many CRCs have a cold phenotype [17]. In 2017, the US Food and Drug Administration (FDA) approved PD-1 immune checkpoint inhibitors pembrolizumab and nivolumab for patients with unresectable or metastatic, mismatch repair deficient (dMMR) and microsatellite instability high (MSI-H) solid tumors who have failed first-line therapy [18, 19]. However, patients with dMMR and MSI-H metastatic CRC (mCRC) comprise only 15% of CRC cases, while the more common mismatch repair proficient (pMMR) and microsatellite stable (MSS) CRC do not respond to ICIs [20]. New strategies are urgently needed for cold mCRCs.

To overcome the hyporesponsiveness to PD-1/PD-L1 inhibitors, recent preclinical studies and clinical trials have demonstrated combination strategies to potentiate the effectiveness of anti-PD-1 and anti-PD-L1 immunotherapy in patients with cold CRC. The FDA has approved combination use of PD-1/PD-L1 inhibitors and other therapy/inhibitors for treatment of patients with cold metastatic cancer. For example, combination of cytotoxic T-lymphocyte-associated protein 4 (CTLA-4) inhibitor, tremelimumab, and PD-1 inhibitor, durvalumab, was approved for treating patients with unresectable hepatocellular carcinoma in 2022 [21, 22]. This review will discuss hyporesponse mechanisms and challenges of PD-1/PD-L1 inhibitors in pMMR/MSS cold cancer and explore potential combination strategies to overcome hyporesponsiveness. Further, we discuss clinical experience with combination therapy and recommendations for future research using CRC as an example.

## Basic mechanisms of hyporesponse in pMMR/MSS cancer

### Carcinogenesis of pMMR/ MSS cancer vs. dMMR/MSI-H cancer

Genomic instability is a trademark of tumor cells. There are two different types of genomic instability: (1) chromosomal instability, which is the consequence of the loss or gain of chromosomes or large chromosomal fragments and is associated with the majority of CRCs, and (2) microsatellite instability (MSI) which is observed in a small fraction of CRCs. [23] Microsatellites are repeated DNA sequences widely dispersed throughout the genome. [24] These repetitive regions are generally associated with higher mutation rates, and replication errors are corrected by the mismatch repair (MMR) system. [25] If there is a deficiency of the MMR system, microsatellites are more prone to replication errors, resulting in MSI. [26] Tumors with dMMR are more likely to be MSI-high (dMMR/MSI-H), while tumor with all tested MMR proteins intact are expected to be MSS or MSI-low (pMMR/MSS). Five microsatellite markers BAT-25, BAT-26, D2S123, D5S346, and D17S250 have been identified; [27] the MSI status of a patient is categorized based on the number of microsatellite markers that demonstrate instability: MSI-H if at least two microsatellite markers show instability; MSI-L (low-frequency MSI) if only one marker show instability; and microsatellite stable (MSS) if there is no instability present among the markers [28]. dMMR/MSI-H CRC comprises 15% of all CRC cases [20]. Growing clinical studies have demonstrated that anti-PD-1 and anti-PD-L1 immunotherapy have positive responses in dMMR/MSI-H cancers but no objective responses in cold pMMR/MSS CRC [6, 7, 15]. There remains a substantial need for novel therapeutic approaches and treatment strategies in metastatic pMMR/MSS CRC.

### Immunogenic features of MSS vs. MSI-H CRC

dMMR/MSI-H CRCs generally have a higher tumor mutational burden (TMB). TMB directly correlates to tumor’s ability to harbor a plethora of neoantigens [29]. Immunogenic neoantigens, in turn, increase anti-tumor immunity by presenting on major-histocompatibility-complex class I molecules (MHC-1) for T cell recognition. The increased neoantigen in dMMR/MSI-H CRCs results in greater abundance of tumor-infiltrating lymphocytes (TIL) and memory T cells; they are described as hot tumors [30, 31]. By comparison, MSS tumors generally produce self-antigens that fail to activate immune response against tumor cells, and increased activation of oncogenic signaling pathways upsurges immunosuppressive cells and cytokines [32]. The loss of peptides involved in antigen processing further dampens the immunogenicity of MSS tumors [33]. As a result, MSS cancer is associated with absent or inadequate T cell infiltration and an immunosuppressive tumor microenvironment (TME); they are described as cold tumors [34]. An escape from immune surveillance and immune attack leads to the absence of clinical response to PD-1/PD-L1 blockades in pMMR/MSS tumors compared to dMMR/MSI-H tumors.

### PD-1 Inhibitors and PD-L1 Inhibitors in clinical application

To date, many anti-PD-1 antibodies (Abs) and anti-PD-L1 Abs have been developed to block PD-1/PD-L1 signaling. Table 1 lists Abs against PD-1 and PD-L1. Anti-PD-1 Abs (nivolumab, pembrolizumab, and cemiplimab) and anti-PD-L1 Abs (atezolizumab, avelumab, and durvalumab) have been approved by FDA for some solid tumor and hematologic cancers. Nivolumab (Opdivo) is the first human IgG4 monoclonal antibody (mAb) against PD-1 approved by the FDA based on the results from CheckMate-037 with advance melanoma patients [35, 36]. Its indications were expanded to squamous non-small-cell lung cancer (NSCLC) and advanced renal cell carcinoma (RCC) in 2015, [36] Hodgkin’s lymphoma [36] and relapsed/refractory metastatic squamous cell cancer of head and neck (SCCHN) in 2016, [36] and small-cell lung cancer (SCLC) patients in 2018 [36]. The FDA approved the anti-PD-1 mAb pembrolizumab and nivolumab as the second-line treatment for patients with dMMR/MSI-H mCRC in 2017 and approved pembrolizumab as the first-line treatment of patients with dMMR/MSI-H mCRC in June 2020 [35, 36].

**Table 1:** Anti-PD-1 and PD-L1 Abs

Name	Targets	Trade or brand name	Antibody class	Company	Phase
Nivolumab	PD-1	OPDIVO, BMS-936558, MDX1106	Humanized IgG4	Bristol-Meyers Squibb	I, II, III
Pembrolizumab	PD-1	Keytruda, MK-3475, Lambrolizumab	Humanized IgG4	Merck	I, Ib, III
Cemiplimab	PD-1	Libtayo, REGN2810	Humanized IgG4	Sanofi	I/II
Camrelizumab	PD-1	(AiRuiKa)(SHR-1210)	Humanized IgG4	Jiangsu HengRui Medicine Co., Ltd	
Pidilizumab	PD-1	CT-011	Humanized IgG1k	Medivation	II
AMP-224	PD-1		Recombinant fusion protein with PD-L2 Fc	AstraZeneca	I
MEDI0680	PD-1	AMP-514	Humanized IgG4κ	Amplimmune; AstraZeneca; MedImmune	I
Spartalizumab	PD-1	PDR001	Humanized IgG4	Novartis	III
Tislelizumab	PD-1	BGB-A317	Humanized IgG4	Novartis	I, II, III
Balstilimab	PD-1	AGEN2034	Humanized IgG4	Agenus	I, II
Atezolizumab	PD- L1	Tecentriq, MPDL3280A	Humanized IgG1	Roche	Ia. I, III
Avelumab	PD- L1	Bavencio, MSB0010718C	Humanized IgG1	Merck, Pfizer	Ib, II
Durvalumab	PD- L1	Imfinzi, MEDI4736	Humanized IgG1	AstraZeneca	II, III
BMS-936559	PD- L1	MDX-1105	Humanized IgG4	Bristol-Myers Squibb	I
Envafolimab	PD- L1	KN 035 and ASC 22	Human IgG1	Alphamab Oncology	II, III
CK-301	PD- L1	Cosibelimab	Humanized IgG1	Checkpoint Therapeutics	I
CS-1001	PD- L1		Humanized IgG	CStone Pharmaceuticals	I, II, III
SHR-1316	PD- L1	HTI-1088	Humanized IgG4	Hengrui Therapeutics	IB, III
CBT-502	PD- L1	TQB-2450	Humanized IgG1	Chia Tai TianQing (CTTQ)	II
BGB-A333	PD- L1		Humanized IgG1-variant	BeiGene	I, II

Most Abs are genetically engineered for high binding specificity and low off-target adverse effects (AEs) [14, 37, 38]. In general, PD-1/PD-L1 blockades exhibit immune-related AEs including colitis and hepatitis, as well as neutropenia, diarrhea, fatigue, stomatitis, and nausea [6, 15, 38–41]. ICIs have fewer severe AEs than traditional chemotherapy [16, 39].

So far, anti-PD-1 and anti-PD-L1 mAb therapies confer significant clinic benefit only in specific patient populations. Specifically, there are almost no objective responses to anti-PD-1 and anti-PD-L1 therapies observed for patients with ‘cold’ tumors such as MSS mCRC. Combatting resistance mechanisms or hyporesponse of the anti-PD-1/PD-L1 therapy remains a challenge.

## New strategies to overcome hyporesponsiveness: combination treatment

The low immunogenic properties of MSS cancer lead to resistance to PD-L1/PD-1 blockade. To enhance clinical response to the PD-1/PD-L1 inhibitors in pMMR/MSS cancer, one promising strategy is to combine with other anti-tumor agents that target different pathways and increase the immunogenicity of the TME, converting cold tumors to hot tumors. It has been demonstrated that inhibition of CTLA-4, vascular endothelial growth factor (VEGF)/VEGF receptor (VEGFR), mitogen-activated protein kinase (MEK), and signal transducer and activator of transcription 3 (STAT3), or treatment with cytotoxic chemotherapy and radiotherapy increases tumor neoantigens, upregulates MHC-1 expression, enhances dendritic cell (DC) antigen presentation and the release of proinflammatory cytokines, increases the activation, infiltration, and killing activities of T cells, and decreases immunosuppressive cells and cytokines. [13, 16, 42–56] A cold pMMR/MSS tumor is subsequently converted into a hot tumor, which can then be targeted by PD-1/PD-L1 blockades to confer anti-tumor immunity. This synergistic anti-tumor effect is a promising avenue of study, and clinical trials investigating these approaches are summarized in Table 2.

**Table 2:** Outcomes of clinical trials and retrospective studies

	Study design	Status	Intervention/time frame	# of Pts (# of pMMR /MSS Pts)	RR, n (%)	DCR, n (%)	Median PFS, mo (95% CI)	Median OS, mo (95% CI)	Potential efficacy biomarker	References
*PD-1/PD-L1 plus CTLA-4 inhibitor*										
NCT02870920	Phase II two arms	Completed/ 24 m	Durvalumab + tremelimumab	119 (117)	0	27 (22.7)	1.8 (1.8–1.9)	6.6 (6.0–7.4)	TMB ≥ 28 CMS2	[57]
*PD-1/PD-L1 plus VEGF inhibitor and chemotherapy*										
NCT01633970	Phase I multi arms	Completed	Atezolizumab + bevacizumab/FOLFOX	23	12 (52)	–	14.1 (8.7–17.1)	–	Increased CD8 + TIL and PD-L1	[58, 59]
NCT03396926	Phase II single arm	Active, not recruiting/48 m	Pembrolizumab + bevacizumab/capecitabine	44(44)	2 (5)	2(5)	4.3	9.6	–	[60]
NCT03721653 (AtezoTRIBE)	Phase II	Active, not recruiting	Atezolizumab + bevacizumab + FoLFOXIRI	142(134)	884(59)	73(34.4)	12.9 (80% CI, 11.9–13.3)	13.6 (80%IC, 12.9–14.4)	TMB-H, high IC	[61]
BACCI/ NCT02873195	Phase II two arms	Active, not recruiting/ 20 m	Atezolizumab + bevacizumab/capecitabine	82 (70)	8.54^a^	–	4..37 (4.07–6.41)	10.55 (8.21 to NA)	Liver Mets	[62]
*PD-1/PD-L1 plus VEGFR inhibitor*										
NCT03406871	Phase I single arm	Completed	Nivolumab + Regorafenib	25 (24)	(33.3)^a^	–	7.9 (2.9-NR)	NR (9.8-NR)	Non-liver metastasis PD-L1 CPS < 1 TMB-H	[63]
NCT04126733	Phase II single arm	completed	Regorafenib + nivolumab	70	5(7.1)	(38.6)	1.8 (1.8–2.4)	11.9 (7.0-not evaluable)	Immune sensitivity biomarkers angiogenetic biomarkers	[64]
NCT03475953	Phase I/II single arm	Recruiting	Avelumab + Regorafenib	43 (43)	0	23 (54)	3.6 (1.8–5.4)	10.8 (5.9-NR)	CD8 + TIL M2-TAM	[65]
NCT03946917	Phase I/II single arm	Active, not recruiting	Toripalimab + Regorafenib	36 (36)	5 (14.9)	13 (36.1)	3	NR	–	[66]
NCT03797326	Phase II two arms	Active, not recruiting	Pembrolizumab + lenvatinib	32 (32)	7 (22)	15 (47)	2.3 (2.0–5.2)	7.5 (3.9-NR)	–	[67]
NCT03912857	Phase II single arm	Unknown	Camrelizumab + apatinib	9 (9)	0	2 (22.2)	1.83 (1.80–1.86)	7.80 (0–17.07)		[68]
–	Retrospective	–	Nivolumab or Pembrolizumab + Regorafenib	18 (18)	0	5 (31)	2	–	Non-liver metastasis	[69]
–	Retrospective	–	Pembrolizumab or camrelizumab or sintilimab or Toripalimab + Regorafenib	23 (23)	0	18 (78.3)	3.1 (2.32–3.89)^b^	–	Non-liver metastasis	[70]
–	Retrospective	–	Toripalimab + Regorafenib	33 (33)	4(12.1)	16 (48.48)	113 days (0–272.1)	NR	Use after second-line treatment Resected primary lesion	[71]
*PD-1/PD-L1 plus MEK inhibitor*										
NCT01988896	Phase I single arm	Completed	Atezolizumab + cobimetinib	84 (62)	6 (10)^a^	26 (31)^b^	1.9 (1.8–2.3)	9.8 (6.2–14.1)	–	[72]
NCT02788279	Phase III three arms	Completed	Atezolizumab + cobimetinib	183 (170)	5(3)^b^	48 (26)	1.91 (1.87–1.97	8.87 (7.00–10.61)	None	[73]
*Combination with PI3K /AKT/mTOR inhibitor*										
NCT03711058	phase I/II	Active, not recruiting	Copanlisib + Nivolumab	54						[74]
*PD-1/PD-L1 plus STAT3 inhibitor*										
NCT02851004	Phase I/II single arm	Terminated	Pembrolizumab + napabucasin	40 (40)	4 (10)	18 (45)	1.6 (1.4–2.1)	7.3 (5.3–11.8)	TMB-H Non-CMS2 tumor POLE mutation Right-sided colon tumor	[75]
*PD-1/PD-L1 inhibitor plus chemotherapy*										
NCT02860546	Phase II single arm	Completed	Nivolumab + trifluridine/tipiracil	18 (18)	0^b^	10 (56)	2.8 (1.8–5.1)	–	–	[76]
NCT02375672	Phase II single arm	Completed	Pembrolizumab + FOLFOX	30 (22)	6 (53)	0 (100)	NR (5.5-NR)	–	–	[77]
*PD-1/PD-L1 inhibitor plus radiotherapy*										
NCT02437071	Phase II single arm	Active, not recruiting	Pembrolizumab + palliative radiotherapy	11 (11)	1 (9)	–	–	–	–	[78]
NCT03005002	Phase I single arm	Completed	Durvalumab/Tremelimumab + yttrium-90	9 (9)	0	0	–	–	–	[79]
NCT02888743	Phase II two arms	Active, not recruiting	Durvalumab/tremelimumab + low-dose or hypofractionated radiotherapy	18 (18)	0	1 (5.5)	–	3.8 (90%CI, 2.3–5.7)	–	[80, 81]
NCT02298946	Phase I single arm	Completed	Pembrolizumab + stereotactic body radiation/ cyclophosphamide	15(4)	0	3 (20)	2.8 (1.2–2.8)	6.0 (2.8–9.6)	–	[82]
NCT03104439	Phase II single arm	Recruiting	Nivolumab + ipilimumab + radiation	40 (40)	3 (7.5)	7 (17.5)	–	–	–	[83]

^a^Outcome for pMMR/MSS colorectal cancer patients analyzed

^b^Due to insufficient or unevaluable tumor samples

^#^-number; CMS2–consensus molecular subtype-2; CPS–combined positive score; CTLA-4–cytotoxic T Lymphocyte Antigen 4; DCR–disease control rate; IC–Immunoscore; MEK–mitogen-activated protein kinase ; MSS–microsatellite stable; ORR–objective response rate; OS–overall survival; epsilon PD-1–programmed death 1; PD-L1–programmed-death-ligand 1; PFS–progression-free survival; PI3K- phosphatidylinositol 3-kinases; pMMR–mismatch repair proficient; POLE–DNA polymerase; Pts–Patients; STAT3–signal transducer and activator of transcription 3; TAM–tumor-associated macrophages; TMB-H–tumor mutational burden high; TIL–tumor-infiltrating lymphocyte; VEGF–vascular endothelial growth factor; VEGFR–vascular endothelial growth factor receptor

### Combination of CTLA-4 and PD-1/PD-L1 inhibitors

CTLA-4 is an immunoglobulin cell surface receptor constitutively expressed on FoxP3 + Treg as well as conventional T cells following activation by T cell receptor (TCR) signaling [43, 84, 85]. CTLA-4 is a negative T cell regulator structurally similar to the second activation receptor CD28 and exhibits shared binding to B7 ligands on antigen presenting cells (APC) [51]. In the TME, the higher affinity of CTLA-4 for B7 ligands outcompetes the co-stimulatory CD28 receptor and depletes CD28 present in the immune synapse [85, 86]. The loss of the second activation signal (B7-CD28) leads to functionally inactivated and hyporesponsive T cells [87]. Hence, CTLA-4 inhibitors directly reduce the competition between CTLA-4 and CD28 for B7 ligands, promoting naïve T cell priming at the draining lymph nodes [44]. The increased CD28-mediated co-stimulation leads to increased effector T cell proliferation and function [43]. CTLA-4 inhibitors have also been shown to decrease Treg-mediated immunosuppression by selectively depleting Treg in the TME [42]. Ipilimumab and tremelimumab are anti-CTLA-4 Abs approved by the FDA [36, 88].

PD-1 and CTLA-4 function on different subsets of T cells, and on T cells at distinct locations and timing during the cancer-immune response [89, 90]. PD-1 is involved in exhaustion mechanisms in the TME and acts in later stages, while CTLA-4 is primarily involved in the lymph nodes and acts early [51, 91]. As such, the dual ICI treatment with anti-PD-1/anti-PD-L1 and anti-CTLA-4 has shown to reverse the upregulation of other immune checkpoints on T cells, which are induced as a compensatory effect by either drug alone [92]. Furthermore, recent studies have found additional anti-tumor effects specific to the dual combination. The combination prevented CD8 + T cell exhaustion and maintained CD8 + T cells in a responsive state with robust killing abilities against tumor cells [93]. This leads to the terminal differentiation of activated effector CD8 + T cells. The dual inhibition also led to a combination-specific increase in T helper type 1 (Th1) cells [93]. Th1 cells mediated anti-tumor activity through increasing CD8 + T cell infiltration, enhancing antibody responses, and exhibiting Th1 specific cytotoxicity against tumor cells [93, 94]. Inhibitors of CTLA-4 such as ipilimumab and tremelimumab are the first ICIs used for treating cancer patients. Currently, the FDA approved the combination treatment with nivolumab and ipilimumab for dMMR/MSI-H mCRC patients who failed in chemotherapy [95]. Clinical trials of the dual ICI in cold pMMR/MSS CRC are ongoing.

A phase II randomized clinical trial (NCT02870920) studying anti-PD-1 (durvalumab) and anti-CTLA-4 (tremelimumab) combination in patients with pMMR/MSS reported that the dual ICI achieves a prolonged median overall survival (mOS) of 6.6 months in pMMR/MSS mCRC patients, but without objective response (OR) and significant improvement in median progression-free survival (mPFS) [57, 96]. Further subgroup analyses showed that the combination increased overall survival (OS) in patients with TMB higher than 28 months, and patients with consensus molecular subtypes (CMS) 2 had improved OS compared to those with CMS 4 [57, 96–98]. The CMS classification system stratifies colorectal cancer into four subtypes based on gene expression profiles: 1) CMS1 is immunogenic, associated with MSI-H; 2) CMS2 is epithelial and canonical; 3) CMS3 is epithelial and metabolic; and 4) CMS4 is mesenchymal [99]. The available clinical study highlights a therapeutic potential for a subset of pMMR/MSS patients and TMB and CMS might be useful stratification biomarkers.

### Combination of VEGF/VEGFR and PD-1/PD-L1 inhibitors

#### Scientific Rationale of VEGF/FEGFR and PD-1/PD-L1 inhibitors

**Fig. 1:**
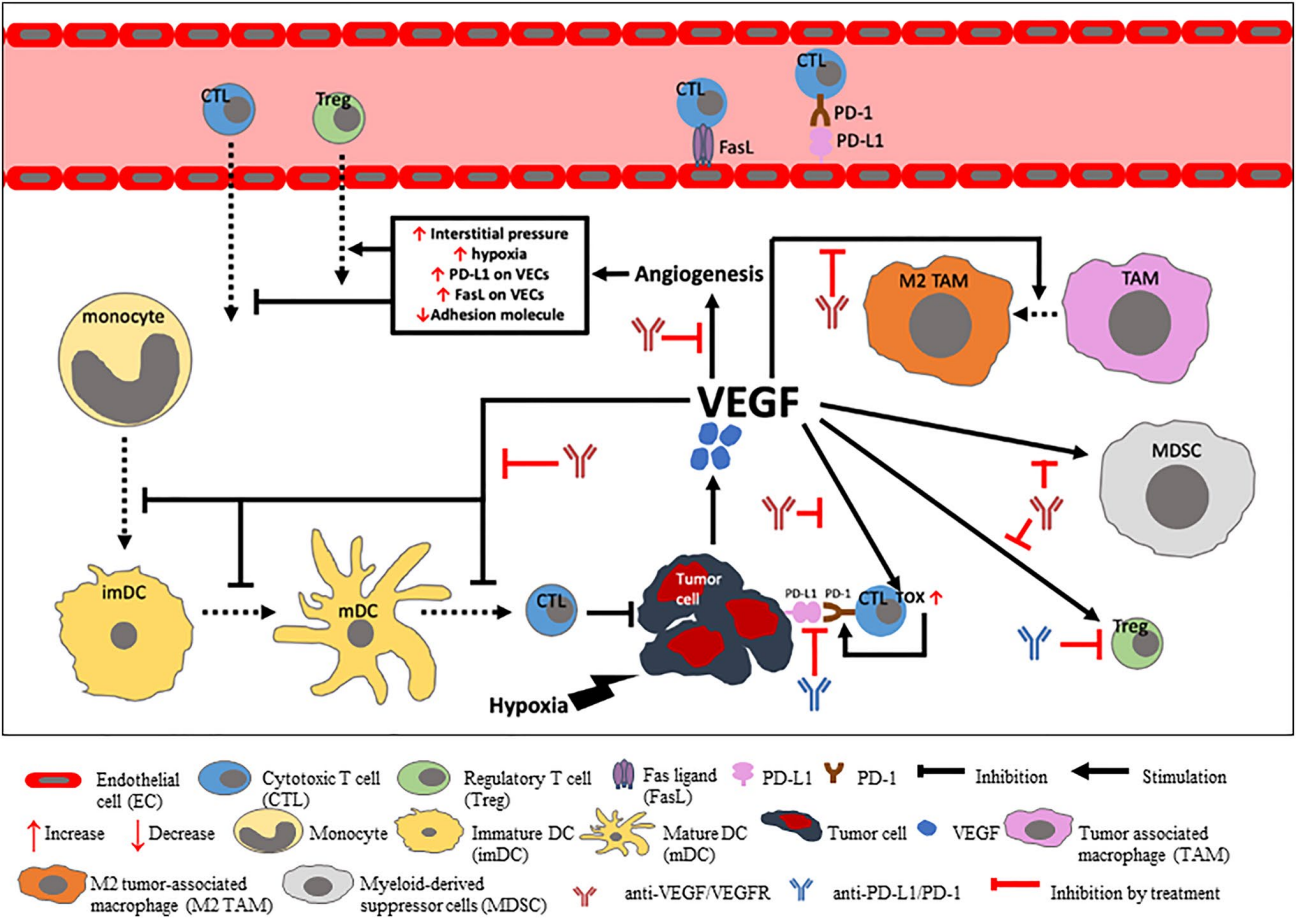
Schematic overview of the role of VEGF in the immunosuppression of the tumor microenvironment (TME)

VEGF/VEGFR signaling plays a vital role in forming the immune-suppressive TME in CRC through indirect and direct pathways (Fig. 1). Overexpression of VEGF/VEGFR signal promotes pathologic angiogenesis, forming highly permeable neovasculature in tumors [100]. The resultant abnormal tumor neovasculature increases fluid accumulation and interstitial fluid pressure in the TME, which acts as a direct barrier against cytotoxic T lymphocyte (CTL) infiltration into tumor tissue [101, 102]. New vessels also differentially express important regulatory molecules involved in anti-tumor immunity. Adhesion molecule downregulation impairs the ability of T cells to move through the vessel walls toward the TME [103, 104]. On the other hand, vascular endothelial cells within the tumor vasculature over-express PD-L1 and Fas ligand (FasL), which induce T cell exhaustion/suppression and selectively kill CTLs, resulting in the predominant infiltration of Treg [105–107]. Furthermore, angiogenesis-mediated hypoxia in the TME increases the expression of chemokines which enhance Treg recruitment and promotes the polarization of tumor-associated macrophages (TAM) to M2-like immunosuppressive phenotype [108, 109]. Beyond angiogenesis, VEGF/VEGFR signaling induces immune suppression by directly acting on immune cells (Fig. 1). VEGF-VEGFR transduction inhibits differentiation, maturation, and antigen presentation of DCs and increases PD-L1 expression on DCs [45, 46, 110, 111]. This leads to reduced naïve CD8 + T cell priming and decreased maintenance of cytotoxic responses against tumors [112]. VEGF also directly inhibits the differentiation of progenitor cells into conventional T cells, decreasing T cell proliferation and cytotoxicity and prompting PD-L1-driven T cell exhaustion [47, 48]. Moreover, it increases the abundance of suppressive or pro-tumor cells such as Treg, myeloid-derived suppressor cell (MDSC), and M2-like immunosuppressive TAM and drives T cell exhaustion [101, 113–115]. Therefore, the inhibition of VEGF/VEGFR signaling could synergistically reduce immune escape to increase the effectiveness of anti-PD-L1/PD-1 inhibitors in patients with cold CRC. Both anti-VEGF therapy and VEGFR tyrosine kinase inhibitors (TKIs) function to inhibit the VEGF signaling pathway. Combination of VEGFR inhibitors (such as regorafenib, lenvatinib, apatinib, and fruquintinib) and PD-1/PD-L1 blockades significantly inhibited angiogenesis and tumor growth in small animal models [116, 117]. The combinations also decreased Treg, shifted macrophages toward M1-like TAM polarization and increased secretion of IFN-γ (an important cytokine involved in tumor.

Tumor cells increase the release of VEGF, which binds to its receptor (VEGFR) to induce angiogenesis. Angiogenesis in turn increases interstitial pressure and hypoxia at the tumor site, which inhibits cytotoxic T cells (CTL) and promotes regulatory T cell (Treg) infiltration. The neovasculature formed via angiogenesis also has higher expression of immunosuppressive molecules PD-L1 and FasL on the vascular endothelial cells (VECs) and lower expression of adhesion molecules. FasL selectively induces CTL apoptosis and PD-L1 inactivates T cells within the tumor vasculature. VEGF/VEGFR also directly modulates immune cell abundance and function. The binding of VEGF to VEGFR inhibits the differentiation and maturation of DCs, which results in reduced T cell activation in the priming phase. It also promotes the proliferation and activation of Tregs and myeloid-derived suppressor cells (MDSCs) and enhances the polarization of tumor-associated macrophages (TAMs) to an M2 phenotype. These immunoregulatory effects reduce CTL function. VEGF also increases the expression TOX in CTL, which in turn upregulates its PD-1 expression and promotes immune exhaustion. Drugs that inhibit VEGF/VEGFR signaling inhibit VEGF/VEGFR-mediated immunosuppression to increase the abundance and function of CTL at the tumor site. Drugs that inhibit PD-L1/PD-1 signaling would block the binding of PD-L1 on CTL to PD-1 on tumor cells and decrease Treg proliferation and function. In combination, anti-VEGF/VEGFR and anti-PD-L1/PD-1 induces a synergistic anti-tumor response immunosurveillance) and overcame PD-L1-induced T cell suppression [118–122]. In fact, positive therapeutic activity has been observed from dual blocking the VEGF/ VEGFR and PD-1/PD-L1 signaling in multiple tumor types [123–127].

#### Combination treatment with PD-L1/PD-1 blockade and anti-VEGF agents

The combination of atezolizumab (anti-PD-L1 Ab) and bevacizumab (VEGF inhibitor) was studied in patients with MSI-H mCRC pretreated with chemotherapy (NCT01633970) and resulted in an objective response rate (ORR) of 30% and a disease control rate of 90%. [128] One clinical study also investigated the efficacy of atezolizumab in combination with bevacizumab and FOLFOX (chemotherapy) in patients with mCRC irrespective of microsatellite status [59]. An ORR of 52% was observed in patients receiving FOLFOX plus bevacizumab and atezolizumab with an mPFS of 14.1 months without unexpected safety signals [59]. The combination significantly elevated tumor-infiltrating CD8 + T cells and PD-L1 expression. Unfortunately, a phase 2 trial studying atezolizumab plus bevacizumab in patients with chemotherapy-resistant, MSI-like CRC** (**NCT02982694) was terminated because the efficacy in the MSS subgroup (MSI like) did not meet the expectation [129]. Subsequently, clinical trials have been focused on triple combination of PD-L1/PD-1 blockade, anti-VEGF agents, and chemotherapy.

AtezoTRIBE (NCT03721653) is a multicenter phase II randomized study for the combination of atezolizumab, bevacizumab, and chemotherapy (FOLFOIXIR) as first-line treatment in patients with unresectable mCRC without prior treatment with chemotherapy [61]. Results show that the combination treatment with atezolizumab did not raise unexpected safety concerns, was well-tolerated and improved PFS. High TMB and high Immunoscore-Immune-Checkpoint (Immunoscore-IC) tumors had better PFS [61].

BACCI (NCT02873195) is a multicenter randomized phase II placebo-controlled clinical trial comparing capecitabine (chemotherapy) and bevacizumab with or without atezolizumab in patients with refractory MSS mCRC [123]. The triple combination resulted in significantly longer mPFS compared to the controlled group in MSS only patients, but did not improve OS (10.55 m v.s 10.61 m in placebo control). The patients without liver metastasis had a higher ORR and greater OS compared with those with liver metastasis, exhibiting synergistic clinical benefits with PD-L1 inhibitor and VEGF inhibition [123].

NCT03396926 is a recent phase II clinical trial evaluating the safety and efficacy of combination capecitabine, bevacizumab and pembrolizumab (anti-PD-1) in locally advanced and metastatic unresectable MSS mCRC patients [60, 130]. To date, the treatment was well-tolerated, and no unexpected safety concerns were reported. About one third of patients had PFS > 6 m, but the ORR was only 5%, not meeting the prespecified target of >  = 15% [130]. However, this study did not include a control group; therefore, it is difficult to draw conclusion on the efficacy of the combination.

Overall, anti-PD-1, atezolizumab, or pembrolizumab, in combination with bevacizumab and chemotherapy, has demonstrated promising results across multiple clinical studies. Exploratory analysis within studies demonstrated that besides MMR status, TMB, Immunoscore-IC, and the presence of liver metastasis are important predictors of treatment outcome. AtezoTRIBE demonstrated improved clinical benefit in patients with high TMB and high Immunoscore-IC, both of which are associated with MSI-H tumor [131, 132]. Cold tumors with low TMB and/or low immunoscore-IC remain a challenge. Pre-screening with these biomarkers or features is necessary to predict clinical outcome of the triple combination treatment.

#### Combination with PD-L1/PD-1 blockade and VEGFR inhibitors

VEGFR inhibitors such as regorafenib, lenvatinib, apatinib, and fruquintinib are studied in combination with PD-L1/PD-1 blockade in patients with pMMR/MSS CRC. The efficacy varied across clinical trials and retrospective studies. Most clinical trials studied the combination of regorafenib and anti-PD-1 mAbs (nivolumab, toripalimab, and pembrolizumab) since regorafenib could enhance T cell activation and increase M1/M2 macrophage ratio compared to inhibitors selective for VEGFR-2 [64, 133].

REGONIVO (NCT03406871, EPOC1603) is a phase Ib/II trial to evaluate regorafenib in combination with anti-PD-1 antibodies nivolumab and toripalimab, respectively, for patients with advanced or metastatic pMMR CRC refractory or intolerant to standard chemotherapy [63]. The results show that 80 mg of regorafenib is optimal in combination with nivolumab, with higher tolerances and fewer toxicities. The study also suggests additional clinical benefits with the combination therapy compared to single agent anti-PD-1, particularly in patients without liver metastasis, CPS < 1, and low TMB [63]. Following the promising findings from the REGONIVO study, the combination of regorafenib and PD-1 inhibitors has been considered as a treatment for refractory pMMR/MSS mCRC patients on a compassionate basis. Two retrospective studies of combination of regorafenib and PD-1 inhibitors (nivolumab or pembrolizumab conducted in the USA, and pembrolizumab, camrelizumab, sintilimab, and toripalimab in China) were conducted in patients with MSS mCRC. No objective responses were reported in patients with the combination therapy, differing from the result of the REGONIVO trial [69, 70]. Consistent with the REGONIVO trial, both retrospective studies suggest that patients with liver metastases do worse despite treatment with regorafenib and anti-PD-1 in pMMR/MSS mCRC. [63, 69, 70] The results of clinical and retrospective studies of regorafenib in combination with anti-PD-1Abs suggest that future investigations of patients with pMMR/MSS mCRC might consider analyzing patients with liver metastases separately, and larger randomized control studies are warranted.

Recent clinical studies with similar combination strategies continued to confer variable results. NCT03946917 is a phase 1b/II study that demonstrated promising results in a subset of unselected pMMR/MSS mCRC patients treated with regorafenib and toripalimab (anti-PD-1) who had progressed or were intolerant to at least 2 prior line of chemotherapy [134]. NCT03712943 is a single-arm phase I of regorafenib plus nivolumab in patients with pMMR mCRC [135]. Fatigue and palmar-plantar erythrodysesthesia, which are frequently associated with the use of regorafenib, were the most common adverse events. Dose limiting toxicity (DLT) was observed. There was no correlation between PD-L1 expression and PFS or OS, but low frequency of Tregs resulted in prolonged PFS. In a multicenter phase 2 trial (NCT04126733) studying combination, regorafenib and nivolumab in patients with pMMR/MSS mCRC demonstrated an ORR of 7%. All patients without liver metastasis responded. Better clinical outcomes may be linked with high expression of pre-existing immune sensitivity biomarkers in tumor samples and lower expression of angiogenetic biomarkers in peripheral blood samples [64]. While treatment outcomes from the combination of regorafenib with anti-PD-1 remain inconsistent, potential benefit may exist in subsets of pMMR/MSS CRC patients.

REGOMUNE (NCT03475953) is the first phase II study that evaluated the efficacy and safety of regorafenib in combination with avelumab (anti-PD-L1) in patients with MSS advanced or metastatic CRC refractory to at least one prior standard therapy [65]. The combination treatment was well-tolerated, and no unexpected adverse events were reported. A significant increase in CD8 + T cell infiltration from baseline was reported in the biomarker analysis comparing tumor samples pre- and post-treatment. The patients with increased CD8 + T cell infiltration had significantly better mPFS and median OS [65]. In contrast to the preliminary biomarker analysis reported in the REGONIVO study, no significant differences in mPFS and median OS were observed in patients with varying PD-L1 expression and TMB status. However, regorafenib and avelumab combination has demonstrated promising impacts on the TME of MSS mCRC patients. The study also reported that high-levels of tumor-infiltrating M2 macrophages prior to the treatment was significantly associated with decreased PFS and OS, suggesting the potential use of tumor-infiltrating M2 macrophage as a predictor for the combination therapy [63, 65]. From the results of the preliminary results, the ongoing REGOMUNE study anticipates further investigation of regorafenib plus anti-PD-1/anti-PD-L1 combination in pMMR/MSS mCRC patients selecting for baseline TAM infiltration levels.

LEAP-005 (NCT03797326) is a recent phase II study evaluating the effectiveness of pembrolizumab and lenvatinib (another oral multi-tyrosine kinase inhibitor of VEGFR) in selected refractory solid tumors including the pMMR/non-MSI-H metastatic and/or unresectable CRC cohort [136]. Promising clinical benefits and a manageable safety profile have been observed in patients with previously treated advanced non-MSI-H/pMMR CRC. Currently, the sample size has been expanded to 100 patients, and the results are anticipated to provide a better understanding of the combination’s effect on anti-tumor activity.

NCT03912857 is a phase II trial of the anti-PD-1 mAb, camrelizumab, in combination with apatinib (a selective tyrosine kinase inhibitor for VEGFR-2) for the treatment of advanced or metastatic MSS CRC refractory to two or more prior lines of standard therapy. [68] Objective response was not reported in the study and intolerable toxicity led to treatment interruptions. In contrast, another study of camrelizumab in combination with apatinib in advanced CRC patients unselected for microsatellite status shows that the ORR in the CRC cohort was 30%. Disease was stable in 80%. Grade 3 and above treatment-related adverse events were observed but manageable. [137] This contrast results also highlighted the potential differences in immunogenicity between MSS and MSI-H mCRC.

Despite the glimpse of a new treatment opportunity for pMMR/MSS mCRC patients brought forward by the REGONIVO study, the results were not replicated in other clinical studies. Nonetheless, the studies suggest the potential use of CD8 + T cell infiltration and low-level TAM2 as a positive predictor for treatment efficacy of regorafenib plus avelumab on the TME [65]. The use of regorafenib and anti-PD-1 recently conferred promising effects as a third-line or later treatment of advanced CRC, especially in patients with resected primary lesions [71]. Thus, combination VEGFR TKIs may open the use of PD-L1/PD-1 inhibitors beyond patients with dMMR/MSI-H mCRC.

### Combination of MEK and PD-1/PD-L1 inhibitors

Mitogen-activated protein kinase (MAPK) cascades are universally conserved transduction pathways that permit extracellular signals to regulate a range of complex physiological cellular programs including cellular proliferation, development, differentiation, migration, survival, and apoptosis [138]. It is well-established that abnormalities in MAPK signal transduction may dysregulate fundamental cellular processes, resulting in cells that acquire the ability to grow uncontrollably and evade apoptosis, leading to tumorigenesis and the progression of cancer [139]. MEK1/ 2 (MAPK kinases) are the only established direct regulators of extracellular signal-regulated kinase 1 (ERK1) and ERK2 and the most well-characterized MAPKs, therefore, play a central role in the Ras-Raf-MEK-ERK cascade [138]. MEK1/2 inhibitors (MEKi) have received attention as a candidate for clinical use in tumors that depend on the ERK pathway [140]. MEKi may also have effects on the immunogenicity of the TME by acting on both tumor and immune cells [49, 50, 72, 141–143]. On tumor cells, MEK can downregulate MHC-I expression [72]. MEKi decrease the secretion of immunosuppressive factors such as VEGF, IL-1, and IL-8, which decreases the recruitment of immunosuppressive cells that inhibit anti-tumor immunity [49]. In addition to tumor cells, MEKi decrease naïve CD8 + T cell priming in the lymph node by preventing MAPK regulation in TCR signaling, while increasing CD8 + T cell infiltration into the TME [50]. MEKi also reduce immunosuppressive cells MDSCs, Tregs, M2-like TAMs, and B-regulatory cells (Breg), which further enhances CD8 + T cell infiltration into the TME [141–143]. Given the immunoinhibitory functions associated with MEK signaling, MEK inhibition could potentially increase TME immunogenicity for the subsequent use of anti-PD-1/anti-PD-L1.

NCT01988896 is a phase Ib clinical study that evaluated the efficacy of cobimetinib (a MEK inhibitor) and atezolizumab in patients with solid tumors, 84 of whom have mCRC [72]. The adverse events observed in the combination treatment were consistent with clinical studies of atezolizumab and cobimetinib monotherapies, but many patients experienced intolerance, which resulted in dose reduction or withdrawal. The objective response of the combination failed to exceed the mPFS and mOS reported in anti-PD-1 monotherapy in MSS mCRC patients. The study suggests that CD8 + T cell infiltration could play a role in tumor response, but was insufficient to induce anti-tumor activity.

Similarly, another phase Ib clinical study (NCT02876224) of atezolizumab and bevacizumab in combination with cobimetinib (MEKi) was conducted in patients with mCRC refractory to one or more lines of prior chemotherapy [144]. They found an ORR of 8%. In a multicenter phase III randomized controlled trial (IMblaze370, NCT02788279) evaluating atezolizumab with cobimetinib in patients with (predominantly) MSS mCRC refractory to two or more lines of chemotherapy, the results show similar OS between the combination and atezolizumab monotherapy and similar mPFS and ORR across all treatment cohorts [73]. No significant differences were demonstrated in PFS and OS between patients with MSS mCRC with different PD-L1 expression and RAS mutation status. More grade 3–4 treatment-related adverse events were reported compared to atezolizumab monotherapy.

The addition of cobimetinib was insufficient to overcome MSS mCRC resistance to atezolizumab. However, potential synergistic activity between MEKi, anti-VEGF, and anti-PD-L1 therapy was observed in the primary analysis of a clinical study described above. Although it is difficult to draw conclusions as to whether the effects were due to the addition of anti-VEGF and/or MEKi, the lack of therapeutic options available for patients with chemo-refractory pMMR/MSS mCRC suggests that a three agent combination strategy is worth exploring.

In addition to MAPK signaling, PI3K/AKT/mTOR signaling is associated with cell survival, migration, division, and other activities. A phase I/II clinical trial (NCT03711058) is currently studying the combination of nivolumab with copanlisib (PI3K inhibitor) in relapsed/refractory pMSS CRC [74].

### Combination of STAT3 and PD-1/PD-L1 inhibitors

STAT3 is an intracellular signaling molecule and transcription factor shown to regulate an array of specific target genes involved in key cellular processes [52]. Sustained activation of STAT3 in tumor cells mediates carcinogenesis through tumor development and growth, angiogenesis, and metastasis [52]. On the other hand, hyperactivation of STAT3 in tumor and immune cells induces immunosuppression and immune evasion [52]. Activation of STAT3 in tumor cells stimulates the release of immunosuppressive factors (e.g., IL-10, VEGF, PD-L1 and indoleamine 2,3-dioxygenase 1) while suppressing proinflammatory cytokines and chemokines [53, 145–147]. Released anti-inflammatory factors in turn activate STAT3 in DCs to prevent DC maturation [148]. With the decrease in antigen presentation by DC, cytotoxic T cells and natural killer cell activation is impeded, and tumor-specific T cell responses are reduced. Therefore, STAT3 inhibition may enhance the activity of anti-PD-L1/anti-PD-1 in patients with MSS mCRC.

Napabucasin is a STAT3 inhibitor studied in combination with anti-PD-1 pembrolizumab in a multicenter phase II clinical trial (NCT02851004) in patients with mCRC refractory or intolerant to at least one regimen of standard chemotherapy [75]. Adverse events associated with the combination of napabucasin and pembrolizumab exhibited safety profiles similar to those observed for either drug alone. The greatest objective response was observed in patients with a higher CPS, and objective response was correlated with an increased TMB. Furthermore, the study found that consensus molecular subtype-2 (CMS2) MSS tumors were more likely to be unresponsive to the combination treatment, while right-sided primary colon cancer was associated with greater clinical benefit [75].

Although primary end point was not met in this clinical trial, napabucasin with pembrolizumab showed greater anti-tumor activity compared to both agents alone. Future studies in a targeted population based on related biomarkers should be further investigated to identify the subset of MSS CRC patients that may receive clinical benefits from the combination therapy.

### Combination cytotoxic chemotherapy and PD-1/PD-L1 inhibitors

Cytotoxic chemotherapy is a fundamental part of treatment for patients with mCRC [149]. Currently, fluorouracil (5-FU) and folinic acid (FA) in combination with oxaliplatin (FOLFOX) or irinotecan (FOLFIRI) is the standard chemotherapy regimen for patients with mCRC [150]. Trifluridine/tipiracil (FTD/TPI) is another chemotherapy combination of trifluridine (a thymidine analog) and tipiracil which inhibits the enzyme involved in trifluridine degradation to maintain bioavailability of trifluridine [151]. Cytotoxic chemotherapy not only kills cancer cells or arrests cancer proliferation, but can also enhance immunogenic effects [54]. Chemotherapies induce more cell death, which triggers the release of tumor-associated antigens (TAA) that are then presented by APC to induce tumor-specific cytotoxic response [152]. On the other hand, chemotherapies could increase ICI expression, introducing rationale for combination with inhibitor of PD-L1/PD-1 signaling [153]. Table S3 lists the immunogenic effects of relevant cytotoxic chemotherapy agents.

A multicenter phase II study (NCT02860546) was conducted in combination of FTD/TPI and nivolumab in patients with chemotherapy-refractory MSS mCRC [76]. The addition of FTD/TPI failed to demonstrate significant potentiation of nivolumab activity, and no objective response was reported. In contrast, another phase II clinical study (NCT02375672) of pembrolizumab and FOLFOX for patients with advanced CRC unselected for MMR status shows that the combination had a promising ORR (53%) in naïve MSS CRC patients with acceptable toxicity [77]. This study suggested that there may be opportunities for chemotherapy-ICI combinations within the context of treating naïve MSS CRC.

As discussed in Sect. 4.2, clinical trials are investigating the potentiation of anti-PD-L1/anti-PD-1 by combining chemotherapy (e.g., FOLFOX, FOLFIRI, FOLFOXFIRI, capecitabine) and anti-VEGF inhibitor (bevacizumab). Promising results from the triple agent regimen have suggested that chemotherapy and anti-VEGF can synergistically modulate the TME to make PD-L1/PD-1 ICI more effective against cold pMMR/MSS CRC [59, 61, 123]. Therefore, both chemotherapy-ICI and chemotherapy/anti-VEGF/ICI are worth exploring for patients with cold mCRC.

### Combination radiotherapy and PD-L1/PD-1 inhibitors

Radiation therapy has been shown to exhibit immune stimulatory effects on the TME via three distinct and overlapping mechanisms: (1) induction of immunogenic cell death (ICD) of tumor cells; (2) upregulation of neoantigen presentation on MHC-1; and (3) direct alteration of the TME at the site of radiation [161]. The ICD induced at the radiation site releases cytokines as well as death-associated molecular patterns (DAMP), which increase the recruitment of DCs and enhance DCs’ ability to phagocytose apoptotic cells and to process and present antigens [55, 161]. This increases T cell priming and infiltration of tumor-specific T cells. Cytokine (Type-I interferons) release further enhances DC stimulation and T cell activation [162]. Radiation also directly upregulates molecules on the surface of tumor cells, which increases the recognition and killing by T cells and NK cells [56]. Beyond the immediate irradiated field, radiotherapy has been shown to induce systemic immunity via abscopal effects [163]. The distinctive immunostimulatory properties of radiotherapy provides a clear rationale for the combination of radiotherapy-anti- PD-1/PD-L1 in patients with MSS mCRC unresponsive to PD-L1/PD-1 blockade alone. Preclinical studies in tumor-bearing mice found that the combination of tumor radiation and anti-PD-L1 synergistically reduced abundance of MDSC within the TME [164].

To date, no significant clinical responses have been observed across four clinical studies in combination with PD-1 inhibitors [79, 80, 82]. There is a phase II study (NCT02437071) evaluating the anti-tumor response at a distant site outside of the irradiated field patients with pMMR mCRC refractory to at least 2 lines of standard therapy treated with pembrolizumab following radiotherapy [165]. Preliminary results reported objective response in 9% of the patients without grade 3 or higher adverse events; therefore, the study continues, and results are anticipated.

One potential approach to improve the efficacy of anti-PD-1 plus radiotherapy in patients with MSS mCRC relies on the use of multiple nonredundant ICIs. In a phase II clinical trial (NCT03104439), MSS mCRC patients refractory to two or more lines of prior therapy received a combination treatment with ipilimumab (anti-CTLA-4) and nivolumab in conjunction with 8 Gy of radiotherapy [83]. The combination of dual ICIs with radiotherapy was feasible and demonstrated durable activity in patients with MSS mCRC. Correlative serial tumor biopsies and updated efficacy results are anticipated. As follow-up, a phase 2 trial of the same regimen is currently enrolling subjects (NCT04575922) [166].

## Future directions

Despite the theoretical framework obtained from preclinical studies of pMMR/MSS cold CRC, limited success was observed across clinical studies for the different combination strategies. Small sample sizes and heterogeneity of tumors or TME in each trial could explain this finding. Comparisons between molecular and cellular phenotypes of common mouse syngeneic models and human tumors may increase our understanding of the mismatched results drawn from preclinical and clinical experiences. Better biomarker detection and patient classification prior to treatment is critical to improve outcomes of combination therapies. Furthermore, it is important to note that oncological signaling pathways (VEGF/VEGF, STAT3, MEK1/2, etc.) have broad biological functions that could be difficult to target specifically or selectively in MSS CRC cells. There are other immune-suppressive molecules or pathways in TME; multiple signaling pathways participate in tumor development and progression. New combinations with other signaling inhibitors or reagents such as temozolomide, which can induce mutation in tumor cells, need to be investigated. We recognize the complexity of the TME; therefore, we suggest future studies to focus on identifying better preclinical models that closely mimic the TME of MSS CRC and efficacy biomarkers in the pMMR/MSS CRC population.

Oncologic outcomes are improving with acceptably safe use of aggressive surgical and local therapy for colorectal liver metastases in carefully selected patients. Evaluating the benefit of systemic immunotherapy either in conjunction with those therapies or following them will be an important avenue for future study. An active multicenter early phase II study is currently investigating the effectiveness of local tumor ablation (radiofrequency ablation or stereotactic body radiation therapy) in combination with durvalumab (Anti-PD-1) and tremelimumab (anti-CTLA-4) in ICI naïve patients with unresectable colorectal liver metastases (NCT03101475). ^168^

## Conclusions

Combination strategies with other anti-tumor agents to potentiate the efficacy of anti-PD-L1/anti-PD-1 in patients with pMMR/MSS advanced or metastatic CRC has become a major research interest as it provides new therapeutic opportunities. In general, combination treatment is safe without significant AEs compared with monotherapy. Preliminary analyses of combination anti-PD-1/PD-L1 inhibitors and other anti-cancer therapies revealed potential clinical benefits in certain subgroups of patients with pMMR/MSS mCRC. Focused approaches to studying these combination regimens will improve outcome of PD-1/PD-L1 combination treatment. We believe that combination strategies involving PD-L1/PD-1 blockade remain a priority for future research as it has the potential to elicit benefits that will revolutionize the clinical landscape for patients with pMMR/MSS cold CRC.

### Supplementary Information

Below is the link to the electronic supplementary material.

Supplementary file1 (DOCX 22 kb)

## Abbreviations

5-FU: Fluorouracil
Ab: Antibody
AE: Adverse effect
APC: Antigen presenting cell
Breg: B-regulatory cell
CPS: Combined positive score
CR: Complete response
CRC: Colorectal Cancer
CTL: Cytotoxic T lymphocytes
CTLA-4: Cytotoxic T lymphocyte antigen 4
DAMP: Death-associated molecular pattern
DC: Dendritic cell
dMMR/MSI-H: Mismatch repair deficient and microsatellite instability high
FA: Folinic acid
Fasl: Fas ligand
FTD: Trifluridine
ICD: Immunogenic cell death
ICI: Immune checkpoint inhibitor
IDO1: Indoleamine 2,3-dioxygenase 1
MAPK: Mitogen-activated protein kinase
mCRC: Metastatic colorectal cancer
MDSC: Myeloid-derived suppressor cell
MEK: Mitogen-activated protein kinase
MHC-1: Major-histocompatibility-complex class I
mPFS: Median progression-free survival
OR: Objective response
ORR: Objective response rate
OS: Overall survival
OX: Oxaliplatin
PD-1: Programmed death 1
PD-L1: Programmed death-ligand 1
PFS: Progression-free survival
pMMR/MSS: Mismatch repair proficient and microsatellite stable
PR: Partial response
STAT3: Signal transducer and activator of transcription 3
TAM: Tumor-associated macrophage
TCR: T cell receptor
TIL: Tumor-infiltrating lymphocyte
TMB: Tumor mutational burden
TME: Tumor microenvironment
TPI: Tipiracil
Treg: Regulatory T cells
VEGF: Vascular endothelial growth factor
VEGFR: Vascular endothelial growth factor receptor

## References

[1] Lugowska I, Teterycz P, Rutkowski P (2018). Immunotherapy of melanoma. Contemp Oncol (Pozn).

[2] Kanwal B, Biswas S, Seminara RS, Jeet C (2018). Immunotherapy in advanced non-small cell lung cancer patients: ushering chemotherapy through the checkpoint inhibitors?. Cureus.

[3] Andre T, Shiu KK, Kim TW, Jensen BV, Jensen LH, Punt C, Smith D, Garcia-Carbonero R, Benavides M, Gibbs P, de la Fouchardiere C, Rivera F, Elez E, Bendell J, Le DT, Yoshino T, Van Cutsem E, Yang P, Farooqui MZH, Marinello P, Diaz LA (2020). Pembrolizumab in microsatellite-instability-high advanced colorectal cancer. N Engl J Med..

[4] Cercek A, Lumish M, Sinopoli J, Weiss J, Shia J, Lamendola-Essel M, El Dika IH, Segal N, Shcherba M, Sugarman R, Stadler Z, Yaeger R, Smith JJ, Rousseau B, Argiles G, Patel M, Desai A, Saltz LB, Widmar M, Iyer K, Zhang J, Gianino N, Crane C, Romesser PB, Pappou EP, Paty P, Garcia-Aguilar J, Gonen M, Gollub M, Weiser MR, Schalper KA, Diaz LA (2022). PD-1 blockade in mismatch repair-deficient, locally advanced rectal cancer. N Engl J Med.

[5] Darvin P, Toor SM, Sasidharan Nair V, Elkord E (2018). Immune checkpoint inhibitors: recent progress and potential biomarkers. Exp Mol Med.

[6] Le DT, Kim TW, Van Cutsem E, Geva R, Jager D, Hara H, Burge M, O'Neil B, Kavan P, Yoshino T, Guimbaud R, Taniguchi H, Elez E, Al-Batran SE, Boland PM, Crocenzi T, Atreya CE, Cui Y, Dai T, Marinello P, Diaz LA, Andre T (2020). Phase II open-label study of pembrolizumab in treatment-refractory, microsatellite instability-high/mismatch repair-deficient metastatic colorectal cancer: KEYNOTE-164. J Clin Oncol.

[7] Overman MJ, McDermott R, Leach JL, Lonardi S, Lenz HJ, Morse MA, Desai J, Hill A, Axelson M, Moss RA, Goldberg MV, Cao ZA, Ledeine JM, Maglinte GA, Kopetz S, Andre T (2017). Nivolumab in patients with metastatic DNA mismatch repair-deficient or microsatellite instability-high colorectal cancer (CheckMate 142): an open-label, multicentre, phase 2 study. Lancet Oncol.

[8] Ishida Y, Agata Y, Shibahara K, Honjo T (1992). Induced expression of PD-1, a novel member of the immunoglobulin gene superfamily, upon programmed cell death. EMBO J.

[9] Agata Y, Kawasaki A, Nishimura H, Ishida Y, Tsubata T, Yagita H, Honjo T (1996). Expression of the PD-1 antigen on the surface of stimulated mouse T and B lymphocytes. Int Immunol.

[10] Pauken KE, Torchia JA, Chaudhri A, Sharpe AH, Freeman GJ (2021). Emerging concepts in PD-1 checkpoint biology. Semin Immunol.

[11] Zhao Y, Harrison DL, Song Y, Ji J, Huang J, Hui E (2018). Antigen-presenting cell-intrinsic PD-1 neutralizes PD-L1 in cis to attenuate PD-1 signaling in T cells. Cell Rep.

[12] Cai J, Wang D, Zhang G, Guo X (2019). The role of PD-1/PD-L1 axis in treg development and function: implications for cancer immunotherapy. Onco Targets Ther.

[13] Jiang Y, Chen M, Nie H, Yuan Y (2019). PD-1 and PD-L1 in cancer immunotherapy: clinical implications and future considerations. Hum Vaccin Immunother.

[14] Philips GK, Atkins M (2015). Therapeutic uses of anti-PD-1 and anti-PD-L1 antibodies. Int Immunol.

[15] O'Neil BH, Wallmark JM, Lorente D, Elez E, Raimbourg J, Gomez-Roca C, Ejadi S, Piha-Paul SA, Stein MN, Abdul Razak AR, Dotti K, Santoro A, Cohen RB, Gould M, Saraf S, Stein K, Han SW (2017). Safety and antitumor activity of the anti-PD-1 antibody pembrolizumab in patients with advanced colorectal carcinoma. PLoS ONE.

[16] Ooki A, Shinozaki E, Yamaguchi K (2021). Immunotherapy in colorectal cancer: current and future strategies. J Anus Rectum Colon.

[17] Ma SX, Li L, Cai H, Guo TK, Zhang LS (2023). Therapeutic challenge for immunotherapy targeting cold colorectal cancer: a narrative review. World J Clin Oncol.

[18] https://www.fda.gov/drugs/resources-information-approved-drugs/fda-grants-nivolumab-accelerated-approval-msi-h-or-dmmr-colorectal-cancer.

[19] https://www.fda.gov/drugs/resources-information-approved-drugs/fda-grants-accelerated-approval-pembrolizumab-first-tissuesite-agnostic-indication;.

[20] Kalyan A, Kircher S, Shah H, Mulcahy M, Benson A (2018). Updates on immunotherapy for colorectal cancer. J Gastrointest Oncol.

[21] https://www.fda.gov/drugs/resources-information-approved-drugs/fda-approves-tremelimumab-combination-durvalumab-unresectable-hepatocellular-carcinoma.

[22] Abou-Alfa GK, Lau George, Kudo Masatoshi, Chan Stephen L, Kelley Robin Kate, Furuse Junji, Sukeepaisarnjaroen Wattana, Yoon-Koo Kang Tu, Dao Van, De Toni Enrico N, Rimassa Lorenza, Breder Valeriy, Vasilyev Alexander, Heurgué Alexandra, Tam Vincent C, Mody Kabir, Thungappa Satheesh Chiradoni, Ostapenko Yuriy, Yau Thomas, Azevedo Sergio, Varela María, Cheng Ann-Lii, Qin Shukui, Galle Peter R, Ali Sajid, Marcovitz Michelle, Makowsky Mallory, He Philip, Kurland John F, Negro Alejandra, Sangro Bruno (2022). Tremelimumab plus durvalumab in unresectable hepatocellular carcinoma. NEJM Evid.

[23] Grady WM (2004). Genomic instability and colon cancer. Cancer Metastasis Rev.

[24] de la Chapelle A, Hampel H (2010). Clinical relevance of microsatellite instability in colorectal cancer. J Clin Oncol.

[25] Li YC, Korol AB, Fahima T, Beiles A, Nevo E (2002). Microsatellites: genomic distribution, putative functions and mutational mechanisms: a review. Mol Ecol.

[26] Jiricny J (2006). The multifaceted mismatch-repair system. Nat Rev Mol Cell Biol.

[27] Losso GM, Moraes Rda S, Gentili AC, Messias-Reason IT (2012). Microsatellite instability–MSI markers (BAT26, BAT25, D2S123, D5S346, D17S250) in rectal cancer. Arq Bras Cir Dig.

[28] Boland CR, Thibodeau SN, Hamilton SR, Sidransky D, Eshleman JR, Burt RW, Meltzer SJ, Rodriguez-Bigas MA, Fodde R, Ranzani GN, Srivastava S (1998). A national cancer institute workshop on microsatellite instability for cancer detection and familial predisposition: development of international criteria for the determination of microsatellite instability in colorectal cancer. Cancer Res.

[29] Llosa NJ, Cruise M, Tam A, Wicks EC, Hechenbleikner EM, Taube JM, Blosser RL, Fan H, Wang H, Luber BS, Zhang M, Papadopoulos N, Kinzler KW, Vogelstein B, Sears CL, Anders RA, Pardoll DM, Housseau F (2015). The vigorous immune microenvironment of microsatellite instable colon cancer is balanced by multiple counter-inhibitory checkpoints. Cancer Discov.

[30] Giannakis M, Mu XJ, Shukla SA, Qian ZR, Cohen O, Nishihara R, Bahl S, Cao Y, Amin-Mansour A, Yamauchi M, Sukawa Y, Stewart C, Rosenberg M, Mima K, Inamura K, Nosho K, Nowak JA, Lawrence MS, Giovannucci EL, Chan AT, Ng K, Meyerhardt JA, Van Allen EM, Getz G, Gabriel SB, Lander ES, Wu CJ, Fuchs CS, Ogino S, Garraway LA (2016). Genomic correlates of immune-cell infiltrates in colorectal carcinoma. Cell Rep.

[31] Wu M, Zheng D, Zhang D, Yu P, Peng L, Chen F, Lin Z, Cai Z, Li J, Wei Z, Lin X, Liu J, Liu X (2020). Converting immune cold into hot by biosynthetic functional vesicles to boost systematic antitumor immunity. Science..

[32] Loddenkemper C, Nagorsen D, Zeitz M (2008). Foxp3 and microsatellite stability phenotype in colorectal cancer. Gut.

[33] Grasso CS, Giannakis M, Wells DK, Hamada T, Mu XJ, Quist M, Nowak JA, Nishihara R, Qian ZR, Inamura K, Morikawa T, Nosho K, Abril-Rodriguez G, Connolly C, Escuin-Ordinas H, Geybels MS, Grady WM, Hsu L, Hu-Lieskovan S, Huyghe JR, Kim YJ, Krystofinski P, Leiserson MDM, Montoya DJ, Nadel BB, Pellegrini M, Pritchard CC, Puig-Saus C, Quist EH, Raphael BJ, Salipante SJ, Shin DS, Shinbrot E, Shirts B, Shukla S, Stanford JL, Sun W, Tsoi J, Upfill-Brown A, Wheeler DA, Wu CJ, Yu M, Zaidi SH, Zaretsky JM, Gabriel SB, Lander ES, Garraway LA, Hudson TJ, Fuchs CS, Ribas A, Ogino S, Peters U (2018). Genetic mechanisms of immune evasion in colorectal cancer. Cancer Discov.

[34] Bonaventura P, Shekarian T, Alcazer V, Valladeau-Guilemond J, Valsesia-Wittmann S, Amigorena S, Caux C, Depil S (2019). Cold tumors: a therapeutic challenge for immunotherapy. Front Immunol.

[35] https://www.fda.gov/drugs/resources-information-approved-drugs/.

[36] Vaddepally RK, Kharel P, Pandey R, Garje R, Chandra AB (2020). Review of indications of FDA-approved immune checkpoint inhibitors per NCCN guidelines with the level of evidence. Cancers..

[37] Castelli MS, McGonigle P, Hornby PJ (2019). The pharmacology and therapeutic applications of monoclonal antibodies. Pharmacol Res Perspect.

[38] Akinleye A, Rasool Z (2019). Immune checkpoint inhibitors of PD-L1 as cancer therapeutics. J Hematol Oncol.

[39] Rajan A, Kim C, Heery CR, Guha U, Gulley JL (2016). Nivolumab, anti-programmed death-1 (PD-1) monoclonal antibody immunotherapy: Role in advanced cancers. Hum Vaccin Immunother.

[40] Baxi S, Yang A, Gennarelli RL, Khan N, Wang Z, Boyce L, Korenstein D (2018). Immune-related adverse events for anti-PD-1 and anti-PD-L1 drugs: systematic review and meta-analysis. BMJ.

[41] Naidoo J, Page DB, Li BT, Connell LC, Schindler K, Lacouture ME, Postow MA, Wolchok JD (2015). Toxicities of the anti-PD-1 and anti-PD-L1 immune checkpoint antibodies. Ann Oncol.

[42] Selby MJ, Engelhardt JJ, Quigley M, Henning KA, Chen T, Srinivasan M, Korman AJ (2013). Anti-CTLA-4 antibodies of IgG2a isotype enhance antitumor activity through reduction of intratumoral regulatory T cells. Cancer Immunol Res.

[43] Syed Khaja AS, Toor SM, El Salhat H, Ali BR, Elkord E (2017). Intratumoral FoxP3(+)Helios(+) regulatory T cells upregulating immunosuppressive molecules are expanded in human colorectal cancer. Front Immunol.

[44] Sotomayor EM, Borrello I, Tubb E, Allison JP, Levitsky HI (1999). In vivo blockade of CTLA-4 enhances the priming of responsive T cells but fails to prevent the induction of tumor antigen-specific tolerance. Proc Natl Acad Sci U S A.

[45] Alfaro C, Suarez N, Gonzalez A, Solano S, Erro L, Dubrot J, Palazon A, Hervas-Stubbs S, Gurpide A, Lopez-Picazo JM, Grande-Pulido E, Melero I, Perez-Gracia JL (2009). Influence of bevacizumab, sunitinib and sorafenib as single agents or in combination on the inhibitory effects of VEGF on human dendritic cell differentiation from monocytes. Br J Cancer.

[46] Curiel TJ, Wei S, Dong H, Alvarez X, Cheng P, Mottram P, Krzysiek R, Knutson KL, Daniel B, Zimmermann MC, David O, Burow M, Gordon A, Dhurandhar N, Myers L, Berggren R, Hemminki A, Alvarez RD, Emilie D, Curiel DT, Chen L, Zou W (2003). Blockade of B7–H1 improves myeloid dendritic cell-mediated antitumor immunity. Nat Med.

[47] Ohm JE, Gabrilovich DI, Sempowski GD, Kisseleva E, Parman KS, Nadaf S, Carbone DP (2003). VEGF inhibits T-cell development and may contribute to tumor-induced immune suppression. Blood.

[48] Voron T, Colussi O, Marcheteau E, Pernot S, Nizard M, Pointet AL, Latreche S, Bergaya S, Benhamouda N, Tanchot C, Stockmann C, Combe P, Berger A, Zinzindohoue F, Yagita H, Tartour E, Taieb J, Terme M (2015). VEGF-A modulates expression of inhibitory checkpoints on CD8+ T cells in tumors. J Exp Med.

[49] Liu L, Mayes PA, Eastman S, Shi H, Yadavilli S, Zhang T, Yang J, Seestaller-Wehr L, Zhang SY, Hopson C, Tsvetkov L, Jing J, Zhang S, Smothers J, Hoos A (2015). The BRAF and MEK inhibitors dabrafenib and trametinib: effects on immune function and in combination with immunomodulatory antibodies targeting PD-1, PD-L1, and CTLA-4. Clin Cancer Res.

[50] Ebert PJR, Cheung J, Yang Y, McNamara E, Hong R, Moskalenko M, Gould SE, Maecker H, Irving BA, Kim JM, Belvin M, Mellman I (2016). MAP kinase inhibition promotes T cell and anti-tumor activity in combination with PD-L1 checkpoint blockade. Immunity.

[51] Brunner-Weinzierl MC, Hoff H, Burmester GR (2004). Multiple functions for CD28 and cytotoxic T lymphocyte antigen-4 during different phases of T cell responses: implications for arthritis and autoimmune diseases. Arthritis Res Ther.

[52] Ferguson SD, Srinivasan VM, Heimberger AB (2015). The role of STAT3 in tumor-mediated immune suppression. J Neurooncol.

[53] Wolfle SJ, Strebovsky J, Bartz H, Sahr A, Arnold C, Kaiser C, Dalpke AH, Heeg K (2011). PD-L1 expression on tolerogenic APCs is controlled by STAT-3. Eur J Immunol.

[54] Opzoomer JW, Sosnowska D, Anstee JE, Spicer JF, Arnold JN (2019). Cytotoxic chemotherapy as an immune stimulus: a molecular perspective on turning up the immunological heat on cancer. Front Immunol.

[55] Krombach J, Hennel R, Brix N, Orth M, Schoetz U, Ernst A, Schuster J, Zuchtriegel G, Reichel CA, Bierschenk S, Sperandio M, Vogl T, Unkel S, Belka C, Lauber K (2019). Priming anti-tumor immunity by radiotherapy: dying tumor cell-derived DAMPs trigger endothelial cell activation and recruitment of myeloid cells. Oncoimmunology.

[56] Reits EA, Hodge JW, Herberts CA, Groothuis TA, Chakraborty M, Wansley EK, Camphausen K, Luiten RM, de Ru AH, Neijssen J, Griekspoor A, Mesman E, Verreck FA, Spits H, Schlom J, van Veelen P, Neefjes JJ (2006). Radiation modulates the peptide repertoire, enhances MHC class I expression, and induces successful antitumor immunotherapy. J Exp Med.

[57] Chen EX, Jonker DJ, Loree JM, Kennecke HF, Berry SR, Couture F, Ahmad CE, Goffin JR, Kavan P, Harb M, Colwell B, Samimi S, Samson B, Abbas T, Aucoin N, Aubin F, Koski SL, Wei AC, Magoski NM, Tu D, O'Callaghan CJ (2020). Effect of combined immune checkpoint inhibition vs best supportive care alone in patients with advanced colorectal cancer: the canadian cancer trials group CO.26 study. JAMA Oncol..

[58] Hochster S, Murphy Janet, Leveque Vincent, Cha Edward, Funke Roel, Waterkamp Daniel, Hegde Priti, Bende Johanna (2016). Clinical activity and immune correlates from a phase Ib study evaluating atezolizumab (anti-PDL1) in combination with FOLFOX and bevacizumab (anti-VEGF) in metastatic colorectal carcinoma. Cancer Res.

[59] Bendell JCPJ, Lieu CH, Eckhardt SG, Hurwitz H, Hochster HS, Murphy JE, Funke RP, Rossi C, Wallin J, Waterkamp D, Pishvaian MJ (2015). Safety and efficacy of MPDL3280A (anti-PDL1) in combination with bevacizumab (bev) and/or FOLFOX in patients (pts) with metastatic colorectal cancer (mCRC). J Clin Oncol.

[60] Bocobo AG, Wang R, Behr S (2021). Phase II study of pembrolizumab plus capecitabine and bevacizumab in microsatellite stable (MSS) metastatic colorectal cancer (mCRC): Interim analysis. J Clin Oncol.

[61] Antoniotti C, Rossini D, Pietrantonio F, Catteau A, Salvatore L, Lonardi S, Boquet I, Tamberi S, Marmorino F, Moretto R, Ambrosini M, Tamburini E, Tortora G, Passardi A, Bergamo F, Kassambara A, Sbarrato T, Morano F, Ritorto G, Borelli B, Boccaccino A, Conca V, Giordano M, Ugolini C, Fieschi J, Papadopulos A, Massoue C, Aprile G, Antonuzzo L, Gelsomino F, Martinelli E, Pella N, Masi G, Fontanini G, Boni L, Galon J, Cremolini C, Investigators GF (2022). Upfront FOLFOXIRI plus bevacizumab with or without atezolizumab in the treatment of patients with metastatic colorectal cancer (AtezoTRIBE): a multicentre, open-label, randomised, controlled, phase 2 trial. Lancet Oncol.

[62] Mettu NB, Ou F-S, Halfdanarson TR, Lenz HJ, Breakstone R, Boland PM, Crysler O, Wu C, Grothey A, Nixon AB, Bolch E, Niedzwiecki D, Fruth B, Schweitzer B, Elsing A, Hurwitz H, Fakih MG, Bekaii-Saab T (2019). BACCI: a phase II randomized, double-blind, multicenter, placebo-controlled study of capecitabine (C) bevacizumab (B) plus atezolizumab (A) or placebo (P) in refractory metastatic colorectal cancer (mCRC)––an ACCRU network study. Ann Oncol.

[63] Fukuoka S, Hara H, Takahashi N, Kojima T, Kawazoe A, Asayama M, Yoshii T, Kotani D, Tamura H, Mikamoto Y, Hirano N, Wakabayashi M, Nomura S, Sato A, Kuwata T, Togashi Y, Nishikawa H, Shitara K (2020). Regorafenib plus nivolumab in patients with advanced gastric or colorectal cancer: an open-label, dose-escalation, and dose-expansion phase Ib trial (REGONIVO, EPOC1603). J Clin Oncol.

[64] Fakih M, Raghav KPS, Chang DZ, Larson T, Cohn AL, Huyck TK, Cosgrove D, Fiorillo JA, Tam R, D'Adamo D, Sharma N, Brennan BJ, Wang YA, Coppieters S, Zebger-Gong H, Weispfenning A, Seidel H, Ploeger BA, Mueller U, Oliveira CSV, Paulson AS (2023). Regorafenib plus nivolumab in patients with mismatch repair-proficient/microsatellite stable metastatic colorectal cancer: a single-arm, open-label, multicentre phase 2 study. EClin Med.

[65] Cousin S, Cantarel C, Guegan JP, Gomez-Roca C, Metges JP, Adenis A, Pernot S, Bellera C, Kind M, Auzanneau C, Le Loarer F, Soubeyran I, Bessede A, Italiano A (2021). Regorafenib-avelumab combination in patients with microsatellite stable colorectal cancer (REGOMUNE): A single-arm, open-label. Phase II Trial Clin Cancer Res.

[66] Wang FHM, Yao Y, Wang Z, Jin Y, Wang FH, Qiu MZ, Lv ZD, Wang DS, Luo HY, Li YH, Zhang DS, Xu R (2020). A phase Ib/II clinical trial of tolerability, safety and efficacy of regorafenib in combination with toripalimab (a PD-1 antibody) in patients with relapsed or metastatic colorectal cancer, 2020. Ann Oncol.

[67] Gomez-Roca Carlos, Eduardo Y, Im Seock-Ah, Alvarez Eduardo Castanon, Senellart Helene, Doherty Mark, García-Corbacho Javier, Lopez Juanita Suzanne, Basu Bristi, Maurice-Dror Corinne, Gill Sanjeev Singh, Ghori Razi, Kubiak Peter, Jin Fan, Norwood Kevin Glen, Chung Hyun Cheol (2021). LEAP-005: a phase II multicohort study of lenvatinib plus pembrolizumab in patients with previously treated selected solid tumors—Results from the colorectal cancer cohort. J Clin Oncol.

[68] Ren C, Mai ZJ, Jin Y, He MM, Wang ZQ, Luo HY, Zhang DS, Wu CY, Wang F, Xu RH (2020). Anti-PD-1 antibody SHR-1210 plus apatinib for metastatic colorectal cancer: a prospective, single-arm, open-label, phase II trial. Am J Cancer Res.

[69] Wang C, Chevalier D, Saluja J, Sandhu J, Lau C, Fakih M (2020). Regorafenib and nivolumab or pembrolizumab combination and circulating tumor DNA response assessment in refractory microsatellite stable colorectal cancer. Oncol.

[70] Li J, Cong L, Liu J, Peng L, Wang J, Feng A, Yue J, Li L, Wang X, Wang X (2020). The efficacy and safety of regorafenib in combination with anti-PD-1 antibody in refractory microsatellite stable metastatic colorectal cancer: a retrospective study. Front Oncol.

[71] Yu W, Tao Q, Zhang Y, Yi F, Feng L (2021). Efficacy and safety of regorafenib combined with toripalimab in the third-line and beyond treatment of advanced colorectal cancer. J Oncol.

[72] Hellmann MD, Kim TW, Lee CB, Goh BC, Miller WH, Oh DY, Jamal R, Chee CE, Chow LQM, Gainor JF, Desai J, Solomon BJ, Das Thakur M, Pitcher B, Foster P, Hernandez G, Wongchenko MJ, Cha E, Bang YJ, Siu LL, Bendell J (2019). Phase Ib study of atezolizumab combined with cobimetinib in patients with solid tumors. Ann Oncol.

[73] Eng C, Kim TW, Bendell J, Argiles G, Tebbutt NC, Di Bartolomeo M, Falcone A, Fakih M, Kozloff M, Segal NH, Sobrero A, Yan Y, Chang I, Uyei A, Roberts L, Ciardiello F, Investigators IM (2019). Atezolizumab with or without cobimetinib versus regorafenib in previously treated metastatic colorectal cancer (IMblaze370): a multicentre, open-label, phase 3, randomised, controlled trial. Lancet Oncol.

[74] Jakubowski CCN, Sugar EA (2020). A phase I/II study of PI3Kinase inhibition with copanlisib combined with the anti-PD-1 antibody nivolumab in relapsed/refractory solid tumors with expansions in MSS colorectal cancer. JCO.

[75] Kawazoe A, Kuboki Y, Shinozaki E, Hara H, Nishina T, Komatsu Y, Yuki S, Wakabayashi M, Nomura S, Sato A, Kuwata T, Kawazu M, Mano H, Togashi Y, Nishikawa H, Yoshino T (2020). Multicenter phase I/II trial of napabucasin and pembrolizumab in patients with metastatic colorectal cancer (EPOC1503/SCOOP Trial). Clin Cancer Res.

[76] Patel MR, Falchook GS, Hamada K, Makris L, Bendell JC (2021). A phase 2 trial of trifluridine/tipiracil plus nivolumab in patients with heavily pretreated microsatellite-stable metastatic colorectal cancer. Cancer Med.

[77] Safi Shahda AMN, Bekaii-Saab Tanios S, O'Neil Bert H, Sehdev Amikar, Shaib Walid Labib, Helft Paul R, Loehrer Patrick J, Tong Yan, Liu Ziyue, El-Rayes Bassel F (2017). A phase II study of pembrolizumab in combination with mFOLFOX6 for patients with advanced colorectal cancer. J Clin Oncol.

[78] Neil Howard Segal NEK, Cercek Andrea, Reidy Diane Lauren, Raasch Pamela Joan, Warren Peter, Hrabovsky Anastasia E, Campbell Naomi, Shia Jinru, Goodman Karyn A, Erinjeri Joseph Patrick, Solomon Stephen Barnett, Yamada Yoshiya, Saltz Leonard (2016). Non-randomized phase II study to assess the efficacy of pembrolizumab (Pem) plus radiotherapy (RT) or ablation in mismatch repair proficient (pMMR) metastatic colorectal cancer (mCRC) patients. J Clin Oncol.

[79] Wang C, Park J, Ouyang C, Longmate JA, Tajon M, Chao J, Lim D, Sandhu J, Yin HH, Pillai R, Gozo MC, Avalos C, Egelston CA, Lee PP, Fakih M (2020). A pilot feasibility study of yttrium-90 liver radioembolization followed by durvalumab and tremelimumab in patients with microsatellite stable colorectal cancer liver metastases. Oncologist.

[80] Monjazeb AG-HA, Lako A, Tesfaye AA, Stroiney A, Gentzler RD, Jabbour S, Alese OB, Rahma OE, Cleary JM, Sharon E, Raben D, Mamon HJ, Streicher H, Chen HX, Ahmed M, Gjini E, Rodig S, Hodi FS, Schoenfeld JD (2019). Analysis of colorectal cancer patients treated on ETCTN 10021: A multicenter randomized trial of combined PD-L1 and CTLA-4 inhibition with targeted low-dose or hypofractionated radiation]. J Clin Oncol.

[81] Monjazeb AM, Giobbie-Hurder A, Lako A, Thrash EM, Brennick RC, Kao KZ, Manuszak C, Gentzler RD, Tesfaye A, Jabbour SK, Alese OB, Rahma OE, Cleary JM, Sharon E, Mamon HJ, Cho M, Streicher H, Chen HX, Ahmed MM, Marino-Enriquez A, Kim-Schulze S, Gnjatic S, Maverakis E, Marusina AI, Merleev AA, Severgnini M, Pfaff KL, Lindsay J, Weirather JL, Ranasinghe S, Spektor A, Rodig SJ, Hodi SF, Schoenfeld JD (2021). A randomized trial of combined PD-L1 and CTLA-4 inhibition with targeted low-dose or hypofractionated radiation for patients with metastatic colorectal cancer. Clin Cancer Res.

[82] Floudas CS, Brar G, Mabry-Hrones D, Duffy AG, Wood B, Levy E, Krishnasamy V, Fioravanti S, Bonilla CM, Walker M, Morelli MP, Kleiner DE, Steinberg SM, Figg WD, Greten TF, Xie C (2019). A pilot study of the PD-1 targeting agent AMP-224 used with low-dose cyclophosphamide and stereotactic body radiation therapy in patients with metastatic colorectal cancer. Clin Colorectal Cancer.

[83] https://clinicaltrials.gov/ct2/show/NCT03104439.

[84] Toor SM, Murshed K, Al-Dhaheri M, Khawar M, Abu Nada M, Elkord E (2019). Immune checkpoints in circulating and tumor-infiltrating CD4(+) T cell subsets in colorectal cancer patients. Front Immunol.

[85] Jain N, Nguyen H, Chambers C, Kang J (2010). Dual function of CTLA-4 in regulatory T cells and conventional T cells to prevent multiorgan autoimmunity. Proc Natl Acad Sci U S A.

[86] Tai X, Van Laethem F, Pobezinsky L, Guinter T, Sharrow SO, Adams A, Granger L, Kruhlak M, Lindsten T, Thompson CB, Feigenbaum L, Singer A (2012). Basis of CTLA-4 function in regulatory and conventional CD4(+) T cells. Blood.

[87] Rochman Y, Yukawa M, Kartashov AV, Barski A (2015). Functional characterization of human T cell hyporesponsiveness induced by CTLA4-Ig. PLoS ONE.

[88] Keam SJ (2023). Tremelimumab: first approval. Drugs.

[89] Seidel JA, Otsuka A, Kabashima K (2018). Anti-PD-1 and Anti-CTLA-4 therapies in cancer: mechanisms of action, efficacy, and limitations. Front Oncol.

[90] Fife B, Bluestone J (2008). Control of peripheral T-cell tolerance and autoimmunity via the CTLA-4 and PD-1 pathways. Immunol Rev.

[91] Huang RY, Francois A, McGray AR, Miliotto A, Odunsi K (2017). Compensatory upregulation of PD-1, LAG-3, and CTLA-4 limits the efficacy of single-agent checkpoint blockade in metastatic ovarian cancer. Oncoimmunology.

[92] Wei SC, Anang NAS, Sharma R, Andrews MC, Reuben A, Levine JH, Cogdill AP, Mancuso JJ, Wargo JA, Pe'er D, Allison JP (2019). Combination anti-CTLA-4 plus anti-PD-1 checkpoint blockade utilizes cellular mechanisms partially distinct from monotherapies. Proc Natl Acad Sci U S A.

[93] Tay RE, Richardson EK, Toh HC (2021). Revisiting the role of CD4(+) T cells in cancer immunotherapy-new insights into old paradigms. Cancer Gene Ther.

[94] https://www.fda.gov/drugs/resources-information-approved-drugs/fda-grants-accelerated-approval-ipilimumab-msi-h-or-dmmr-metastatic-colorectal-cancer.

[95] Overman MJ, Lonardi S, Wong KYM, Lenz HJ, Gelsomino F, Aglietta M, Morse MA, Van Cutsem E, McDermott R, Hill A, Sawyer MB, Hendlisz A, Neyns B, Svrcek M, Moss RA, Ledeine JM, Cao ZA, Kamble S, Kopetz S, Andre T (2018). Durable clinical benefit with nivolumab plus ipilimumab in DNA mismatch repair-deficient/microsatellite instability-high metastatic colorectal cancer. J Clin Oncol.

[96] Lote H, Starling N, Pihlak R, Gerlinger M (2022). Advances in immunotherapy for MMR proficient colorectal cancer. Cancer Treat Rev.

[97] Jonathan M, Loree JTT, Kennecke Hagen Fritz, Feilotter Harriet, Lee Young S, Virk Shakeel, Kopetz Scott, Duose Dzifa Yawa, Manyam Ganiraju C, Morris Jeffrey S, Maru Dipen M, Renouf Daniel, Jonker Derek J, Dongsheng Tu, O'Callaghan Christopher J, Chen Eric Xueyu (2022). Impact of consensus molecular subtyping (CMS) on survival in the CO.26 trial of durvalumab plus tremelimumab versus best supportive care (BSC) in metastatic colorectal cancer (mCRC). J Clin Oncol.

[98] Guinney J, Dienstmann R, Wang X, de Reynies A, Schlicker A, Soneson C, Marisa L, Roepman P, Nyamundanda G, Angelino P, Bot BM, Morris JS, Simon IM, Gerster S, Fessler E, De Sousa EMF, Missiaglia E, Ramay H, Barras D, Homicsko K, Maru D, Manyam GC, Broom B, Boige V, Perez-Villamil B, Laderas T, Salazar R, Gray JW, Hanahan D, Tabernero J, Bernards R, Friend SH, Laurent-Puig P, Medema JP, Sadanandam A, Wessels L, Delorenzi M, Kopetz S, Vermeulen L, Tejpar S (2015). The consensus molecular subtypes of colorectal cancer. Nat Med.

[99] Falcon BL, O'Clair B, McClure D, Evans GF, Stewart J, Swearingen ML, Chen Y, Allard K, Lee LN, Neote K, McEwen DP, Uhlik MT, Chintharlapalli S (2013). Development and characterization of a high-throughput in vitro cord formation model insensitive to VEGF inhibition. J Hematol Oncol.

[100] Rahma OE, Hodi FS (2019). The intersection between tumor angiogenesis and immune suppression. Clin Cancer Res.

[101] Lee WS, Yang H, Chon HJ, Kim C (2020). Combination of anti-angiogenic therapy and immune checkpoint blockade normalizes vascular-immune crosstalk to potentiate cancer immunity. Exp Mol Med.

[102] Lanitis E, Irving M, Coukos G (2015). Targeting the tumor vasculature to enhance T cell activity. Curr Opin Immunol.

[103] Fisher DT, Chen Q, Skitzki JJ, Muhitch JB, Zhou L, Appenheimer MM, Vardam TD, Weis EL, Passanese J, Wang WC, Gollnick SO, Dewhirst MW, Rose-John S, Repasky EA, Baumann H, Evans SS (2011). IL-6 trans-signaling licenses mouse and human tumor microvascular gateways for trafficking of cytotoxic T cells. J Clin Invest.

[104] Barsoum IB, Smallwood CA, Siemens DR, Graham CH (2014). A mechanism of hypoxia-mediated escape from adaptive immunity in cancer cells. Cancer Res.

[105] Chen J, Jiang CC, Jin L, Zhang XD (2016). Regulation of PD-L1: a novel role of pro-survival signalling in cancer. Ann Oncol.

[106] Zhang W, Ding EX, Wang Q, Zhu DQ, He J, Li YL, Wang YH (2005). Fas ligand expression in colon cancer: a possible mechanism of tumor immune privilege. World J Gastroenterol.

[107] Facciabene A, Peng X, Hagemann IS, Balint K, Barchetti A, Wang LP, Gimotty PA, Gilks CB, Lal P, Zhang L, Coukos G (2011). Tumour hypoxia promotes tolerance and angiogenesis via CCL28 and T(reg) cells. Nature.

[108] Movahedi K, Laoui D, Gysemans C, Baeten M, Stange G, Van den Bossche J, Mack M, Pipeleers D, In't Veld P, De Baetselier P, Van Ginderachter JA (2010). Different tumor microenvironments contain functionally distinct subsets of macrophages derived from Ly6C(high) monocytes. Cancer Res.

[109] Oyama T, Ran S, Ishida T, Nadaf S, Kerr L, Carbone DP, Gabrilovich DI (1998). Vascular endothelial growth factor affects dendritic cell maturation through the inhibition of nuclear factor-kappa B activation in hemopoietic progenitor cells. J Immunol.

[110] Gabrilovich DI, Chen HL, Girgis KR, Cunningham HT, Meny GM, Nadaf S, Kavanaugh D, Carbone DP (1996). Production of vascular endothelial growth factor by human tumors inhibits the functional maturation of dendritic cells. Nat Med.

[111] Fu C, Jiang A (2018). Dendritic cells and CD8 T cell immunity in tumor microenvironment. Front Immunol.

[112] Terme M, Pernot S, Marcheteau E, Sandoval F, Benhamouda N, Colussi O, Dubreuil O, Carpentier AF, Tartour E, Taieb J (2013). VEGFA-VEGFR pathway blockade inhibits tumor-induced regulatory T-cell proliferation in colorectal cancer. Cancer Res.

[113] Yang J, Yan J, Liu B (2018). Targeting VEGF/VEGFR to modulate antitumor immunity. Front Immunol.

[114] Kim CG, Jang M, Kim Y, Leem G, Kim KH, Lee H, Kim TS, Choi SJ, Kim HD, Han JW, Kwon M, Kim JH, Lee AJ, Nam SK, Bae SJ, Lee SB, Shin SJ, Park SH, Ahn JB, Jung I, Lee KY, Park SH, Kim H, Min BS, Shin EC (2019). VEGF-A drives TOX-dependent T cell exhaustion in anti-PD-1-resistant microsatellite stable colorectal cancers. Sci Immunol..

[115] Doleschel D, Hoff S, Koletnik S, Rix A, Zopf D, Kiessling F, Lederle W (2021). Regorafenib enhances anti-PD1 immunotherapy efficacy in murine colorectal cancers and their combination prevents tumor regrowth. J Exp Clin Cancer Res.

[116] Fiegle E, Doleschel D, Koletnik S, Rix A, Weiskirchen R, Borkham-Kamphorst E, Kiessling F, Lederle W (2019). Dual CTLA-4 and PD-L1 blockade inhibits tumor growth and liver metastasis in a highly aggressive orthotopic mouse model of colon cancer. Neoplasia.

[117] Zhou K, Zhang JW, Wang QZ, Liu WY, Liu JL, Yao L, Cai MM, Ni SY, Cai QY, Wang GJ, Zhou F (2019). Apatinib, a selective VEGFR2 inhibitor, improves the delivery of chemotherapeutic agents to tumors by normalizing tumor vessels in LoVo colon cancer xenograft mice. Acta Pharmacol Sin.

[118] Wang Y, Wei B, Gao J, Cai X, Xu L, Zhong H, Wang B, Sun Y, Guo W, Xu Q, Gu Y (2020). Combination of fruquintinib and anti-PD-1 for the treatment of colorectal cancer. J Immunol.

[119] Cai X, Wei B, Li L, Chen X, Liu W, Cui J, Lin Y, Sun Y, Xu Q, Guo W, Gu Y (2020). Apatinib enhanced anti-PD-1 therapy for colon cancer in mice via promoting PD-L1 expression. Int Immunopharmacol.

[120] Wang Q, Gao J, Di W, Wu X (2020). Anti-angiogenesis therapy overcomes the innate resistance to PD-1/PD-L1 blockade in VEGFA-overexpressed mouse tumor models. Cancer Immunol Immunother.

[121] Kato Y, Tabata K, Kimura T, Yachie-Kinoshita A, Ozawa Y, Yamada K, Ito J, Tachino S, Hori Y, Matsuki M, Matsuoka Y, Ghosh S, Kitano H, Nomoto K, Matsui J, Funahashi Y (2019). Lenvatinib plus anti-PD-1 antibody combination treatment activates CD8+ T cells through reduction of tumor-associated macrophage and activation of the interferon pathway. PLoS ONE.

[122] Mettu NB, Ou FS, Zemla TJ, Halfdanarson TR, Lenz HJ, Breakstone RA, Boland PM, Crysler OV, Wu C, Nixon AB, Bolch E, Niedzwiecki D, Elsing A, Hurwitz HI, Fakih MG, Bekaii-Saab T (2022). Assessment of capecitabine and bevacizumab with or without atezolizumab for the treatment of refractory metastatic colorectal cancer: a randomized clinical trial. JAMA Netw Open.

[123] Finn RS, Qin S, Ikeda M, Galle PR, Ducreux M, Kim TY, Kudo M, Breder V, Merle P, Kaseb AO, Li D, Verret W, Xu DZ, Hernandez S, Liu J, Huang C, Mulla S, Wang Y, Lim HY, Zhu AX, Cheng AL, Investigators IM (2020). Atezolizumab plus bevacizumab in unresectable hepatocellular carcinoma. N Engl J Med.

[124] Socinski MA, Jotte RM, Cappuzzo F, Orlandi F, Stroyakovskiy D, Nogami N, Rodriguez-Abreu D, Moro-Sibilot D, Thomas CA, Barlesi F, Finley G, Kelsch C, Lee A, Coleman S, Deng Y, Shen Y, Kowanetz M, Lopez-Chavez A, Sandler A, Reck M, Group IMS (2018). Atezolizumab for first-line treatment of metastatic nonsquamous NSCLC. N Engl J Med..

[125] Rini BI, Powles T, Atkins MB, Escudier B, McDermott DF, Suarez C, Bracarda S, Stadler WM, Donskov F, Lee JL, Hawkins R, Ravaud A, Alekseev B, Staehler M, Uemura M, De Giorgi U, Mellado B, Porta C, Melichar B, Gurney H, Bedke J, Choueiri TK, Parnis F, Khaznadar T, Thobhani A, Li S, Piault-Louis E, Frantz G, Huseni M, Schiff C, Green MC, Motzer RJ, Group IMS (2019). Atezolizumab plus bevacizumab versus sunitinib in patients with previously untreated metastatic renal cell carcinoma (IMmotion151): a multicentre, open-label, phase 3, randomised controlled trial. Lancet..

[126] Rini BI, Plimack ER, Stus V, Gafanov R, Hawkins R, Nosov D, Pouliot F, Alekseev B, Soulieres D, Melichar B, Vynnychenko I, Kryzhanivska A, Bondarenko I, Azevedo SJ, Borchiellini D, Szczylik C, Markus M, McDermott RS, Bedke J, Tartas S, Chang YH, Tamada S, Shou Q, Perini RF, Chen M, Atkins MB, Powles T (2019). Pembrolizumab plus axitinib versus sunitinib for advanced renal-cell carcinoma. N Engl J Med..

[127] Hochster HS, Bendell JC, Cleary JM, Foster P, Zhang W, He X, Hernandez G, Iizuka K, Eckhardt SG (2017). Efficacy and safety of atezolizumab (atezo) and bevacizumab (bev) in a phase Ib study of microsatellite instability (MSI)-high metastatic colorectal cancer (mCRC). J Clin Oncol.

[128] https://clinicaltrials.gov/ct2/show/NCT02982694.

[129] Bocobo AGWR, Behr S (2022). Phase II study of pembrolizumab plus capecitabine and bevacizumab in microsatellite stable (MSS) metastatic colorectal cancer (mCRC). JCO.

[130] Zhou Z, Xie X, Wang X, Zhang X, Li W, Sun T, Cai Y, Wu J, Dang C, Zhang H (2021). Correlations between tumor mutation burden and immunocyte infiltration and their prognostic value in colon cancer. Front Genet.

[131] Noepel-Duennebacke S, Juette H, Schulmann K, Graeven U, Porschen R, Stoehlmacher J, Hegewisch-Becker S, Raulf A, Arnold D, Reinacher-Schick A, Tannapfel A (2021). J Cancer Res Clin Oncol..

[132] Ou DL, Chen CW, Hsu CL, Chung CH, Feng ZR, Lee BS, Cheng AL, Yang MH, Hsu C (2021). Regorafenib enhances antitumor immunity via inhibition of p38 kinase/Creb1/Klf4 axis in tumor-associated macrophages. J Immunother Cancer..

[133] Wang F, He M, Yao Y, Wang Z, Jin YK, Wang F, Qiu M, Lv Z, Wang D, Luo H, Li Y, Zhang D, Xu R (2020). 433P A phase Ib/II clinical trial of tolerability, safety and efficacy of regorafenib in combination with toripalimab (a PD-1 antibody) in patients with relapsed or metastatic colorectal cancer. Ann Oncol.

[134] Kim RD, Kovari BP, Martinez M, Xie H, Sahin IH, Mehta R, Strosberg J, Imanirad I, Ghayouri M, Kim YC, Kim DW (2022). A phase I/Ib study of regorafenib and nivolumab in mismatch repair proficient advanced refractory colorectal cancer. Eur J Cancer.

[135] Carlos Gomez-Roca EY, Im Seock-Ah, Alvarez Eduardo Castanon, Senellart Helene, Doherty Mark, García-Corbacho Javier, Lopez Juanita Suzanne, Basu Bristi, Maurice-Dror Corinne, Gill Sanjeev Singh, Ghori Razi, Kubiak Peter, Jin Fan, Norwood Kevin Glen, Chung Hyun Cheol (2021). LEAP-005: a phase II multicohort study of lenvatinib plus pembrolizumab in patients with previously treated selected solid tumors—results from the colorectal cancer cohort. J Clin Oncol.

[136] Xiao LZY, Lin Q (2020). 442P Camrelizumab combined with apatinib in the treatment of patients with advanced gastric cancer and colorectal cancer: one-arm exploratory clinical trial. Ann Oncol.

[137] Wortzel I, Seger R (2011). The ERK cascade: distinct functions within various subcellular organelles. Genes Cancer.

[138] Dhillon AS, Hagan S, Rath O, Kolch W (2007). MAP kinase signalling pathways in cancer. Oncogene.

[139] Fremin C, Meloche S (2010). From basic research to clinical development of MEK1/2 inhibitors for cancer therapy. J Hematol Oncol.

[140] Allegrezza MJ, Rutkowski MR, Stephen TL, Svoronos N, Perales-Puchalt A, Nguyen JM, Payne KK, Singhal S, Eruslanov EB, Tchou J, Conejo-Garcia JR (2016). Trametinib drives T-cell-dependent control of KRAS-mutated tumors by inhibiting pathological myelopoiesis. Cancer Res.

[141] Baumann D, Hagele T, Mochayedi J, Drebant J, Vent C, Blobner S, Noll JH, Nickel I, Schumacher C, Boos SL, Daniel AS, Wendler S, Volkmar M, Strobel O, Offringa R (2020). Proimmunogenic impact of MEK inhibition synergizes with agonist anti-CD40 immunostimulatory antibodies in tumor therapy. Nat Commun.

[142] Yarchoan M, Mohan AA, Dennison L, Vithayathil T, Ruggieri A, Lesinski GB, Armstrong TD, Azad NS, Jaffee EM (2019). MEK inhibition suppresses B regulatory cells and augments anti-tumor immunity. PLoS ONE.

[143] Bendell JLC, Raghav KPS (2019). A phase Ib study of the safety and efficacy of atezolizumab (atezo) + bevacizumab (bev) + cobimetinib (cobi) in patients (pts) with metastatic colorectal cancer (mCRC). Ann Oncol.

[144] Kujawski M, Kortylewski M, Lee H, Herrmann A, Kay H, Yu H (2008). Stat3 mediates myeloid cell-dependent tumor angiogenesis in mice. J Clin Invest.

[145] Wang Y, Shen Y, Wang S, Shen Q, Zhou X (2018). The role of STAT3 in leading the crosstalk between human cancers and the immune system. Cancer Lett.

[146] Litzenburger UM, Opitz CA, Sahm F, Rauschenbach KJ, Trump S, Winter M, Ott M, Ochs K, Lutz C, Liu X, Anastasov N, Lehmann I, Hofer T, von Deimling A, Wick W, Platten M (2014). Constitutive IDO expression in human cancer is sustained by an autocrine signaling loop involving IL-6, STAT3 and the AHR. Oncotarget.

[147] Melillo JA, Song L, Bhagat G, Blazquez AB, Plumlee CR, Lee C, Berin C, Reizis B, Schindler C (2010). Dendritic cell (DC)-specific targeting reveals Stat3 as a negative regulator of DC function. J Immunol.

[148] Gustavsson B, Carlsson G, Machover D, Petrelli N, Roth A, Schmoll HJ, Tveit KM, Gibson F (2015). A review of the evolution of systemic chemotherapy in the management of colorectal cancer. Clin Colorectal Cancer.

[149] Munker S, Gerken M, Fest P, Ott C, Schnoy E, Fichtner-Feigl S, Wiggermann P, Vogelhuber M, Herr W, Stroszczynski C, Schlitt HJ, Evert M, Reng M, Klinkhammer-Schalke M, Teufel A (2018). Chemotherapy for metastatic colon cancer: no effect on survival when the dose is reduced due to side effects. BMC Cancer.

[150] Mayer RJ, Van Cutsem E, Falcone A, Yoshino T, Garcia-Carbonero R, Mizunuma N, Yamazaki K, Shimada Y, Tabernero J, Komatsu Y, Sobrero A, Boucher E, Peeters M, Tran B, Lenz HJ, Zaniboni A, Hochster H, Cleary JM, Prenen H, Benedetti F, Mizuguchi H, Makris L, Ito M, Ohtsu A (2015). Randomized trial of TAS-102 for refractory metastatic colorectal cancer. N Engl J Med..

[151] Cremolini C, Antoniotti C, Rossini D, Lonardi S, Loupakis F, Pietrantonio F, Bordonaro R, Latiano TP, Tamburini E, Santini D, Passardi A, Marmorino F, Grande R, Aprile G, Zaniboni A, Murgioni S, Granetto C, Buonadonna A, Moretto R, Corallo S, Cordio S, Antonuzzo L, Tomasello G, Masi G, Ronzoni M, Di Donato S, Carlomagno C, Clavarezza M, Ritorto G, Mambrini A, Roselli M, Cupini S, Mammoliti S, Fenocchio E, Corgna E, Zagonel V, Fontanini G, Ugolini C, Boni L, Falcone A, Investigators GF (2020). Upfront FOLFOXIRI plus bevacizumab and reintroduction after progression versus mFOLFOX6 plus bevacizumab followed by FOLFIRI plus bevacizumab in the treatment of patients with metastatic colorectal cancer (TRIBE2): a multicentre, open-label, phase 3, randomised, controlled trial. Lancet Oncol.

[152] Dosset M, Vargas TR, Lagrange A, Boidot R, Vegran F, Roussey A, Chalmin F, Dondaine L, Paul C, Lauret Marie-Joseph E, Martin F, Ryffel B, Borg C, Adotevi O, Ghiringhelli F, Apetoh L (2018). PD-1/PD-L1 pathway: an adaptive immune resistance mechanism to immunogenic chemotherapy in colorectal cancer. Oncoimmunology.

[153] Vincent J, Mignot G, Chalmin F, Ladoire S, Bruchard M, Chevriaux A, Martin F, Apetoh L, Rebe C, Ghiringhelli F (2010). 5-Fluorouracil selectively kills tumor-associated myeloid-derived suppressor cells resulting in enhanced T cell-dependent antitumor immunity. Cancer Res.

[154] Wu Y, Deng Z, Wang H, Ma W, Zhou C, Zhang S (2016). Repeated cycles of 5-fluorouracil chemotherapy impaired anti-tumor functions of cytotoxic T cells in a CT26 tumor-bearing mouse model. BMC Immunol.

[155] Duffy AG, Greten TF (2014). Immunological off-target effects of standard treatments in gastrointestinal cancers. Ann Oncol.

[156] Liu WM, Fowler DW, Smith P, Dalgleish AG (2010). Pre-treatment with chemotherapy can enhance the antigenicity and immunogenicity of tumours by promoting adaptive immune responses. Br J Cancer.

[157] Tesniere A, Schlemmer F, Boige V, Kepp O, Martins I, Ghiringhelli F, Aymeric L, Michaud M, Apetoh L, Barault L, Mendiboure J, Pignon JP, Jooste V, van Endert P, Ducreux M, Zitvogel L, Piard F, Kroemer G (2010). Immunogenic death of colon cancer cells treated with oxaliplatin. Oncogene.

[158] Melichar B, Touskova M, Vesely P (2002). Effect of irinotecan on the phenotype of peripheral blood leukocyte populations in patients with metastatic colorectal cancer. Hepatogastroenterology.

[159] Kim HS, Park HM, Park JS, Sohn HJ, Kim SG, Kim HJ, Oh ST, Kim TG (2010). Dendritic cell vaccine in addition to FOLFIRI regimen improve antitumor effects through the inhibition of immunosuppressive cells in murine colorectal cancer model. Vaccine.

[160] Suzuki N, Tsukihara H, Nakagawa F, Kobunai T, Takechi T (2017). Synergistic anticancer activity of a novel oral chemotherapeutic agent containing trifluridine and tipiracil in combination with anti-PD-1 blockade in microsatellite stable-type murine colorectal cancer cells. Am J Cancer Res.

[161] Lim JY, Gerber SA, Murphy SP, Lord EM (2014). Type I interferons induced by radiation therapy mediate recruitment and effector function of CD8(+) T cells. Cancer Immunol Immunother.

[162] Demaria S, Ng B, Devitt ML, Babb JS, Kawashima N, Liebes L, Formenti SC (2004). Ionizing radiation inhibition of distant untreated tumors (abscopal effect) is immune mediated. Int J Radiat Oncol Biol Phys.

[163] Deng L, Liang H, Burnette B, Beckett M, Darga T, Weichselbaum RR, Fu YX (2014). Irradiation and anti-PD-L1 treatment synergistically promote antitumor immunity in mice. J Clin Invest.

[164] https://clinicaltrials.gov/ct2/history/NCT02437071?V_9=View

[165] https://clinicaltrials.gov/ct2/show/NCT04575922

[166] https://clinicaltrials.gov/ct2/show/NCT03101475

