# Supplementary material for: PD-1 and PD-L1 inhibitors in cold colorectal cancer: challenges and strategies
Source: Cancer Immunol Immunother. 2023 Oct 13;72(12):3875–93. doi: 10.1007/s00262-023-03520-5 (PMC10700246; doi:10.1007/s00262-023-03520-5)
Supplement: Supplementary file 1 — Supplementary file1 (DOCX 22 kb) [file 262_2023_3520_MOESM1_ESM.docx]

Table 3 Immunogenic effects of common cytotoxic chemotherapies studied in combination with PD-1/PD-L1 inhibitors in pMMR/MSS CRCs

| Cytotoxic chemotherapy | Immunogenic effects | References |
| --- | --- | --- |
| 5-FU | Selective depletion of MDSCs | [154] |
|  | ↑ CD8+ TILs | [155] |
| OX | ↑ DC function | [156, 157] |
|  | Immunogenic cell death 🡪 ↑ Antigen presentation | [158] |
| Irinotecan | ↑ CD4+ and CD8+ cells | [159] |
|  | ↓ Tregs and MDSCs | [160] |
| Trifluridine | ↑ CD8+ T cell/lymphocyte ratio | [161] |
|  | ↓ Treg/CD4+ T cell ratio | [161] |

PD-1 – Programed Death 1; PD-L1 – Programed-Death Ligand 1; pMMR – mismatch repair proficient; MSS – microsatellite stable; 5-FU – 5-fluorouracil; MDSC – myeloid-derived suppressor cell; TIL – tumor infiltrating lymphocytes, OX – oxaliplatin; DC – dendritic cells; Treg – regulatory T cells
